# Neuro-semantic graph fusion for explainable depression risk trajectory mapping using a graph-enhanced RoBERTa framework

**DOI:** 10.3389/frai.2026.1854179

**Published:** 2026-08-05

**Authors:** M. Karthiga, Emerson Raja Joseph, Subhash Patil, K. Saranya

**Affiliations:** 1 Department of Computer Science and Engineering, Bannari Amman Institute of Technology, Sathyamangalam, Tamilnadu, India; 2 Faculty of Engineering and Technology Centre for Advanced Analytics, COE of Artificial Intelligence, Multimedia University, Melaka, Malaysia; 3 Surgery Department, International Medical University, Management and Science University, Shah Alam, Malaysia

**Keywords:** affective computing, depression risk trajectory, explainable AI, graph attention networks, mental health screening, Roberta, supervised contrastive learning

## Abstract

**Introduction:**

Early identification of depression through social media analytics is frequently compromised by linguistic ambiguities, contextual complexities, and the opaque nature of conventional deep learning architectures. This study proposes Graph-RoBERTa-CL, a hybrid neuro-semantic framework that explicitly integrates three synergistic components: (i) RoBERTa-based Transformer semantic encoding for deep contextual representation, (ii) Graph Attention Networks (GAT) for relational contextual aggregation across semantically related posts, and (iii) Supervised Contrastive Learning (SCL) for latent space regularization under class imbalance, collectively enabling explainable depression risk trajectory mapping from social media corpora.

**Methods:**

The framework was trained and validated on the Suicide Detection Dataset (*N* = 232,074 posts; 78,541 unique users) using rigorous user-level data splitting to ensure zero user overlap between training and test sets, with cross-dataset consistency assessed on an independent Combined Mental Health Dataset (*N* = 3,900 posts) withheld entirely from training and model selection. Both datasets are drawn from Reddit communities with community-based labels; broader generalizability across platforms, populations, and clinically diagnosed cohorts requires further validation. User posts are represented as nodes in a multi-relational semantic graph, where a GAT selectively aggregates contextual embeddings to capture ruminative behavioral patterns and temporal trajectories. A supervised contrastive objective simultaneously reshapes the latent space to improve class separability under linguistic ambiguity, and SHAP-based explanations alongside graph attention visualizations provide structural transparency aligned with established psycholinguistic theories of depression.

**Results:**

On the internal user-level test set, Graph-RoBERTa-CL achieved Accuracy 91.2%, Precision 89.7%, Recall 93.1%, F1-Score 0.914, and AUC 0.962, substantially outperforming BERT-base (F1: 0.798, AUC: 0.934) and LSTM-Attention (F1: 0.681, AUC: 0.856). On the external independent validation set, the model achieved F1 0.882 and AUC 0.941, with the modest cross-dataset reduction confirming genuine generalizability rather than dataset-specific overfitting. Ablation studies confirm that each architectural component — semantic encoding, graph attention aggregation, and contrastive optimization — contributes independently and synergistically to overall performance.

**Discussion:**

The proposed framework establishes a rigorous, interpretable, and ethically grounded mechanism for proactive mental health screening in digital ecosystems, designed to function as a human-in-the-loop clinical screening aid rather than a standalone diagnostic tool.

## 1 Introduction

Depression is one of the most prevalent mental health disorders worldwide, with early identification playing a critical role in preventing severe outcomes such as suicidal ideation and functional impairment. The increasing use of social media platforms has created a rich, albeit noisy, source of user-generated textual data that reflects emotional states, cognitive patterns, and behavioural trajectories. As a result, automated depression risk prediction from social media text has emerged as an important research direction, complementing traditional clinical screening approaches (Amanat et al., 2022; Qasim et al., 2025).

Early studies in this domain primarily relied on conventional machine learning models and lexicon-based features, which were limited in their ability to capture long-range dependencies and subtle emotional cues embedded in natural language (Aldhyani et al., 2022; Amanat et al., 2022). The introduction of deep learning, particularly recurrent and convolutional neural networks, significantly improved performance by learning distributed representations of text; however, these models still struggled with interpretability and contextual ambiguity (Malhotra and Jindal, 2020; Verma et al., 2022). More recently, Transformer-based architectures have become the dominant paradigm due to their superior ability to model contextual semantics and long-term dependencies (Haque et al., 2020; Mandal et al., 2023; Naseem et al., 2020).

Pretrained language models such as BERT and RoBERTa have demonstrated strong performance in depression and suicide ideation detection tasks (Liu et al., 2019; Meng, 2024; Qasim et al., 2025; Xin and Zakaria, 2024). Domain-adapted variants, including MentalBERT, further improve sensitivity to psychological language by leveraging mental health–specific corpora (Ji et al., 2022; Shwetha and Pushpalatha, 2025). Despite these advances, most Transformer-based approaches treat social media posts as independent textual units, ignoring the fact that emotional expression on social platforms is inherently contextual, influenced by semantic similarity, topic continuity, and temporal progression (Go et al., 2009; Wang et al., 2022). To address this limitation, recent research has explored graph-based learning for mental health analysis, modelling relationships among users, posts, or emotional states (Ahmed et al., 2023; Chen et al., 2022; Lu et al., 2022; Mao and Han, 2025). Graph neural networks (GNNs), particularly Graph Attention Networks, enable selective aggregation of contextual information and have shown promise in capturing relational dependencies in mental healthcare data (Ahmed et al., 2023; Lu et al., 2022). However, existing graph-based approaches often rely on shallow textual features or static graph constructions, limiting their ability to fully exploit deep semantic representations learned by large language models.

In parallel, contrastive learning has emerged as a powerful paradigm for representation learning, enabling models to construct well-separated latent spaces even under limited supervision or severe class imbalance (Gao et al., 2021; Obaido et al., 2025). Recent studies have demonstrated the effectiveness of contrastive learning in emotion recognition, medical classification, and multimodal affect analysis (Fan et al., 2024; Gao et al., 2021; Kokilepersaud et al., 2023; Le et al., 2023; Lin et al., 2023; Liu et al., 2023; Obaido et al., 2025; Pan et al., 2022; Zheng et al., 2024). Nevertheless, the application of supervised contrastive learning to depression risk prediction from social media—particularly in conjunction with graph-based contextual modelling—remains underexplored.

In parallel with social media–based analysis, machine learning has been widely explored in clinical psychiatry for predicting treatment response and remission in depressive disorders. Studies have demonstrated the effectiveness of ensemble learning and predictive modelling for antidepressant response using clinical and biological features (Kautzky et al., 2021; Lin et al., 2020; Zhou et al., 2021). While these approaches operate in structured clinical settings, they highlight the broader potential of machine learning to support decision-making in mental health care. Social media–based risk prediction frameworks can be viewed as complementary tools, offering early, non-invasive indicators that precede formal clinical evaluation.

Furthermore, the deployment of AI systems in mental health contexts necessitates explainability, robustness, and ethical accountability. Explainable AI (XAI) methods have been increasingly incorporated to improve trust and clinical interpretability (Hameed et al., 2025; Kerz et al., 2023). Yet, many existing approaches provide post-hoc explanations without explicitly modelling how contextual relationships influence predictions, limiting their usefulness for real-world decision support.

Motivated by these gaps, this work proposes Graph-RoBERTa-CL, a neuro-semantic graph fusion framework that integrates Transformer-based semantic encoding, graph attention–driven contextual aggregation, and supervised contrastive learning to enable explainable depression risk trajectory mapping from social media text. The task is presented as depression risk screening instead of a classification of suicidality, because it uses a psycholinguistic framework that captures the markers of high-severity depressive cognition, a component of the DSM-5 diagnosis of depression, and provides a continuous risk score that is intended to be used in clinical triage, not diagnostic labels. This framing is consistent with the traditional public health screening approach in which items related to suicidal ideation are part of a larger depression severity score, rather than being used as standalone targets for screening. By jointly modelling semantic neighbourhoods and latent space structure, the proposed approach moves beyond isolated text classification toward a clinically informed, context-aware risk assessment paradigm. The core novelty and specific contributions of this research are summarized as follows:

- A unified framework is introduced that synergizes the deep semantic extraction capabilities of RoBERTa with the relational aggregation strengths of Graph Attention Networks (GAT) to capture both individual sentiment and social reinforcement signals.
- A Supervised Contrastive Learning (SCL) objective is implemented to mathematically reshape the latent space, pulling at-risk representations into tight, distinct clusters to overcome the challenges of severe class imbalance in social media data.
- Structural transparency is established through the use of SHAP-based linguistic markers and graph attention weight visualizations, allowing for a clinically aligned interpretation of how specific social contexts influence risk escalation.
- A longitudinal risk monitoring methodology utilizing temporal windowing to detect meaningful behavioural shifts and early clinical markers of depression.
- A rigorous two-phase evaluation: internal user-level evaluation on the Suicide Detection Dataset (F1: 0.914, AUC: 0.962) and external cross-dataset validation on an independent Combined Mental Health Dataset (F1: 0.882, AUC: 0.941), demonstrating genuine generalizability.

## 2 Related works

### 2.1 Transformer-based depression and suicide detection

Transformer-based models have become the foundation of modern depression detection systems due to their ability to capture deep contextual semantics. Studies such as Qasim et al. (2025) and Xin and Zakaria (2024) demonstrated that fine-tuned BERT variants significantly outperform traditional deep learning models. Haque et al. (2020) and Verma et al. (2022) further explored Transformer architectures for suicidal ideation detection, highlighting their robustness across diverse social media platforms. Domain-specific models, including MentalBERT (Ji et al., 2022), were introduced to improve sensitivity to psychological language, while recent evaluations confirmed the superiority of RoBERTa over earlier Transformer variants (Liu et al., 2019; Meng, 2024). Despite strong performance, these approaches primarily operate at the single-post level, failing to capture inter-post dependencies and contextual reinforcement patterns that characterize sustained depressive expression.

Recent studies have emphasized the importance of modelling emotional dynamics and robustness in mental health–oriented language understanding. Paz-Arbaizar et al. demonstrated that transformer-based emotion forecasting can capture temporal affective trends rather than static emotional states, highlighting the importance of longitudinal modelling in psychological assessment (Paz-Arbaizar et al., 2025). In parallel, robustness-enhancing mechanisms for Transformers, including dynamic attention adaptation and perturbation-aware learning, have been proposed to improve stability under noisy or ambiguous linguistic inputs (Kitada and Iyatomi, 2021; Shen et al., 2023). These works underscore the need for architectures that not only achieve high predictive accuracy but also maintain consistent behavior when faced with metaphorical, artistic, or emotionally ambiguous language commonly observed in social media.

Most existing depression and suicide ideation detection studies focus on a limited set of social media platforms, which may restrict generalizability across user demographics and communication styles. Darade investigated suicidal ideation detection using Instagram posts, demonstrating that linguistic expression and visual–textual interaction patterns vary significantly across platforms (Darade, 2023). This observation motivates the use of heterogeneous datasets and platform-agnostic modelling strategies when designing social media–based mental health screening systems.

### 2.2 Graph-based modelling in mental health analysis

Graph-based learning has been explored as a means to capture relational dependencies in mental healthcare data. Ahmed et al. (2023) introduced graph attention–based curriculum learning for mental health classification, while Lu et al. (2022) and Chen et al. (2022) demonstrated the utility of graph structures for predictive risk modelling and mental state classification. Mao and Han (2025) enhanced TextGCN with emotional representations for depression detection, achieving improved contextual reasoning.

However, most graph-based approaches rely on shallow or static text representations, limiting their ability to leverage the expressive power of large pretrained language models. Moreover, few studies integrate graph reasoning with temporal risk aggregation or explainability mechanisms.

Beyond unimodal text analysis, recent research has explored the integration of graph structures and multimodal data for mental health screening. Mo et al. introduced a graph-based anxiety screening framework that models node correlations across multimodal signals, demonstrating the value of relational reasoning in mental health assessment (Mo et al., 2025). Similarly, Jiahui proposed a multimodal deep representation framework for psychological stress inference, highlighting how combining heterogeneous features improves robustness and generalization (Jiahui, 2025). Sensor fusion–based mental engagement analysis further illustrates the effectiveness of integrating multiple data modalities to infer latent cognitive and emotional states (Aminosharieh Najafi et al., 2023). While these studies validate the promise of graph and multimodal approaches, they often lack deep semantic language modelling or supervised contrastive optimization, limiting their applicability to large-scale social media text.

### 2.3 Contrastive learning for emotion and mental health modelling

Contrastive learning has gained traction in affective computing and medical AI for learning robust representations under limited supervision. SimCSE (Gao et al., 2021) laid the foundation for contrastive sentence embedding, while subsequent studies extended contrastive learning to emotion recognition and medical classification (Fan et al., 2024; Gao et al., 2021; Kokilepersaud et al., 2023; Le et al., 2023; Lin et al., 2023; Liu et al., 2023; Obaido et al., 2025; Pan et al., 2022; Zheng et al., 2024). Surveys by Obaido et al. (2025) and Liu et al. (2023) highlight the growing relevance of contrastive learning in biomedical AI.

Despite its success, contrastive learning has rarely been applied to depression risk prediction from social media, particularly in a supervised setting that explicitly structures class-specific latent spaces. The combination of contrastive objectives with graph-based semantic aggregation remains largely unexplored.

Contrastive learning has increasingly been adopted in clinical and textual domains to improve representation discriminability and robustness. Kokilepersaud et al. demonstrated that clinically labelled contrastive learning enhances biomarker classification by enforcing semantically meaningful embedding separation (Kokilepersaud et al., 2023). Similarly, Lin et al. showed that contrastive objectives significantly improve multi-label text classification by mitigating label co-occurrence ambiguity and latent overlap (Lin et al., 2023). These findings motivate the integration of supervised contrastive learning into depression risk prediction, where class imbalance and semantic ambiguity are persistent challenges. However, its combination with graph-based contextual aggregation for mental health analysis remains largely unexplored.

### 2.4 Explainability and clinical Trust in Mental Health AI

Explainability is essential for the adoption of AI systems in mental healthcare. Kerz et al. (2023) and Hameed et al. (2025) emphasized the importance of interpretable language-based indicators for depression detection, while recent surveys highlighted ethical considerations and responsible AI deployment (Chaudhry et al., 2024; Muetunda et al., 2025). Nonetheless, many existing systems provide explanations detached from the underlying model structure, limiting their clinical interpretability.

### 2.5 Recent advances in LLM-based and explainable depression detection

The current studies are experiencing a paradigm shift to Large Language Models (LLMs) and improved explainability of depression detection systems. Shah et al. (2025) showed that GPT-3.5 Turbo and LLaMA2-7B models with task-specific fine-tuning (96.4 percent accuracy) are able to detect content that depicts depression in social media more often than base models can. This paper indicates that instruction-tuned LLMs demonstrate the possibilities of representing subtle language patterns of depression that cannot be done by conventional BERT-based frameworks. In the same way, Liu et al. (2024) suggested a hybrid system of ChatGPT to provide responses, BERT to classify the content, and SHAP to be understandable in the context of depression interventions with a high accuracy of 93.76 and natural human-like dialogue.

This has added to a greater focus on explainability, and several recent papers have made clinical interpretability and predictive performance more important. In Al Masud et al. (2025), SHAP and LIME are used to explain the predictions of Random Forest and RoBERTa on the data of Bangladeshi university students, and their accuracy and recall at the same time are 91.1 and 98.6, respectively. Their publication makes the case of open machine learning in mental health scenarios critical. Hameed et al. (2025) combined LIME with black-box models (SVM, Random Forest, XGBoost, ANN) to detect depression on social media, and the most accurate model with the highest accuracy was SVM. More importantly, they proved that the explanations produced by LIME are consistent with formulated psychological markers, and they bridge the gap between computational predictions and clinical knowledge.

Symptom-based detection has become one of the important methods of enhancing clinical trust. Bao et al. (2024) proposed transformer-based architectures, which are able to both identify depression and provide an explanation of the prediction based on validated symptom markers, with good classification and a natural language based explanation that would be in accordance with clinical requirements. Their dual task strategy of training different models of classification and explanation compared to integrating the two tasks is a valuable methodological development. In addition to that, they investigated in-context learning and symptom-oriented refinement of conversational LLMs, which proved that modern language models are flexible enough to be applied in the clinical field.

The adoption of conversational AI in the implementation of interpretability has been on the rise. Belcastro et al. (2025) suggested BERT-XDD, an explicatory model that combines BERTweet with masked attention, and they used ChatGPT to convert technical explanations into commentaries readable to a human. This anthropocentric way of interpretability is a new step in achieving access to AI predictions among non-technical medical practitioners. Likewise, Jo et al. (2024) used LIME and SHAP to explain predictions of the Random Forest model (99.30% accuracy) on datasets of mental health conditions to explore how depression correlates with comorbid disorders (anxiety, bipolar disorder, and substance use disorders).

Although these have been advanced, there are still a number of limitations. To begin with, the majority of recent literature still regards social media posts as self-contained texts, disregarding the presence of semantic neighbourhoods and time continuity that define the development of prolonged depressive expression. Even the post processing-based approaches of the LLM do not model the relationships. Second, both SHAP and LIME offer post-hoc explanations, but do not connect with the internal logical procedure of a model, which restricts their application to how contextual associations manipulate forecasting. Third, the temporal risk monitoring, which is essential in early intervention, is not a significant concern of recent work, and they instead concentrate on snapshot classification as opposed to longitudinal behavioural change detection.

The proposed Graph-RoBERTa-CL framework bridges these gaps through incorporating semantic graph modelling, supervised contrastive learning and multi-level explainability (SHAP to linguistic markers, graph attention weights to contextual reasoning and temporal trajectories to behavioural shifts). In contrast to recent studies based on LLM, which mainly use increased model size and prompting to learn, our system uses structural inductive biases (graph attention on semantic neighbourhoods) to learn social patterns of reinforcement that play a key role in differentiating transient emotional expression and enduring risk of depression.

### 2.6 Research gap and contributions

Even though Transformer-based models have made beats in terms of detecting depressive posts on social media, it remains the case that the majority of current models, such as the latest studies, assume that posts are discrete units of text, without taking into account the semantic neighbourhoods or temporal continuity that define persistent expression of depressive affect. State-of-the-art LLCM-based systems model single posts without reflecting the relational relations and social reinforcement patterns distinguishing temporal emotional language and long-term depression danger. This weakness lowers clinical accuracy, especially in the process of differentiating between dark humor, sarcasm, and metaphorical utterances and veritable signals of distress. Most importantly, however, traditional Transformer pipelines are not structurally capable of learning about semantically neighboring interactions, such as reinforcement and attenuation signals, arising in the context of a post when semantically close discourse is contributed by the same user. This is not addressed by using a larger model size, nor by using pretraining on the domain specific data; both of these work within the isolated-inference paradigm; it requires an explicit modelling mechanism that works across posts and not within each one individually.

Although recent developments have achieved a lot on explainability using SHAP, LIME and symptom-based explanations (Bao et al., 2024; Belcastro et al., 2025; Hameed et al., 2025), existing XAI methods are largely post-hoc and not tied to internal model reasoning. Graph-based approaches have been investigated to capture the contextual relationship, but most of them involve shallow text representations or fixed graph structures, and more recent development in LLM depression detection does not yet build graph-based arguments. Furthermore, the temporal risk modelling, which is important in identifying any changes of behavior and providing an opportunity to act at the earliest and prevent the issue, is still a relatively unexplored area in recent research, which is mostly concerned with snapshots classification and not yet with a long-term study.

The proposed Graph-RoBERTa-CL framework addresses these limitations through an integrated neuro-semantic modelling strategy. By combining Transformer-based semantic encoding with graph attention–driven contextual aggregation, the framework captures semantic reinforcement and attenuation across related posts, enabling more reliable risk estimation beyond isolated text analysis. To mitigate class imbalance and latent ambiguity, supervised contrastive learning is incorporated to enforce structured embedding spaces, improving class separability and recall for high-risk cases. Explainability is embedded within the architecture through linguistic attribution and graph attention visualization, ensuring transparent and clinically interpretable predictions. Finally, temporal aggregation of risk scores enables depression risk trajectory mapping, transforming static classification into a longitudinal screening tool suitable for early detection and continuous monitoring. A comparative analysis of the proposed Graph-RoBERTa-CL framework against established methodologies in the domain is presented in Table 1. This comparison highlights the transition from isolated textual analysis to a hybrid neuro-semantic approach that incorporates structural context and contrastive optimization.

**Table 1:** Comparative analysis of the proposed methodology and related works.

Ref. no	Proposed methodology	Dataset used	Accuracy obtained	Pros	Cons
Qasim et al. (2025)	Fine-tuned BERT variants	Social media text	High (Relative to traditional DL)	Superior at capturing deep contextual semantics compared to CNN/RNN.	Operates at a single-post level; ignores inter-post dependencies.
Ji et al. (2022)	MentalBERT (domain-adapted transformer)	Mental Health-specific Corpora	High Sensitivity	Improved sensitivity to psychological language and domain-specific vocabulary.	Lacks structural context modelling; treats posts as independent units.
Ahmed et al. (2023)	Graph attention-based curriculum learning	Mental Healthcare Classification data	Improved contextual reasoning	Effectively models relational dependencies in mental health data.	Often relies on shallow or static text representations.
Mao and Han (2025)	Enhanced TextGCN with Emotional Representation	Social Media Depression data	Improved over standard GCN	Better contextual reasoning through emotional feature integration.	Fails to fully leverage the expressive power of large pretrained LLMs.
Gao et al. (2021)	SimCSE (Unsupervised Contrastive Learning)	Sentence Embeddings	Robust Representations	Foundation for robust representation learning with limited supervision.	Rarely applied to specific clinical depression risk prediction tasks.
Shah et al. (2025)	Fine-tuned GPT-3.5 & LLaMA2-7B	Social Media Data	96.4%	Strong performance through task-specific LLM fine-tuning; captures nuanced depressive language patterns.	Treats posts independently; lacks graph-based contextual modelling and temporal trajectory analysis.
Liu et al. (2024)	ChatGPT + BERT + SHAP for Intervention	Depression Intervention Scenarios	93.76%	Integrates LLM response generation with interpretability; maintains conversational quality.	Post-hoc explainability disconnected from internal reasoning; no temporal monitoring capability.
Al Masud et al. (2025)	Random Forest + RoBERTa + SHAP/LIME	Bangladeshi University Students	91.1% (RF), 98.6% Recall (RoBERTa)	Combines ML and DL with comprehensive XAI; transparent decision-making through LIME/SHAP.	Independent post processing; lacks structural context modelling and longitudinal risk assessment.
Hameed et al. (2025)	SVM + Advanced NLP + LIME	Social Media Text	High (SVM Best)	Clinical alignment of LIME explanations with psychological markers; multiple NLP techniques integrated.	Black-box models with post-hoc XAI only; no graph-based reasoning or temporal pattern detection.
Bao et al. (2024)	Transformer + Symptom Markers (Dual-task)	Social Media Depression Data	Good Classification + Explanation	Symptom-based explanations aligned with clinical criteria; unified classification and explanation.	Processes posts individually; missing semantic neighbourhood modelling and behavioural shift tracking.
Belcastro et al. (2025)	BERT-XDD + ChatGPT Explanations	Social Media (Twitter)	Not Reported	Human-readable explanations via ChatGPT; masked attention for self-explanation; accessible to non-technical users.	Lacks graph-based contextual aggregation; no supervised contrastive learning or temporal monitoring.
Jo et al. (2024)	Random Forest + LIME/SHAP	Mental Health Conditions Dataset	99.30%	Extremely high accuracy; explores comorbidity correlations; dual XAI approach (LIME + SHAP).	Traditional ML on structured data; not optimized for noisy social media text; no temporal or graph modelling.
Proposed	Graph-RoBERTa-CL (User-Level Evaluation)	Multi-source Community-Labelled Social Media Dataset	91.2% (Accuracy), 0.914 (F1-Score), 0.962 (AUC)	Captures semantic reinforcement via graph attention; solves class imbalance via SCL; provides longitudinal risk trajectories; integrated multi-level XAI (SHAP + graph attention).	Currently optimized for textual data; generalizability to visual-heavy platforms is still being validated.

## 3 Proposed methodology

### 3.1 Methodological overview and novel contributions

In this paper, we introduce a hybrid neuro-semantic framework called Graph-RoBERTa-CL that explicitly combines Semantic Embedding from the RoBERTa model, Graph Attention Networks (GAT), and Supervised Contrastive Learning (SCL) for explainable depression risk trajectory mapping from social media corpora. Rather than treating each post as a standalone input, the framework models three properties jointly: semantic depth through Transformer-based contextual encoding; semantic neighbourhood and social reinforcement through graph attention; and latent space regularization through supervised contrastive learning to address class overlap and imbalance. The result is a system that detects ruminative behavioural patterns across a user’s posting history rather than isolated keyword signals in a single post.

**Figure 1:**
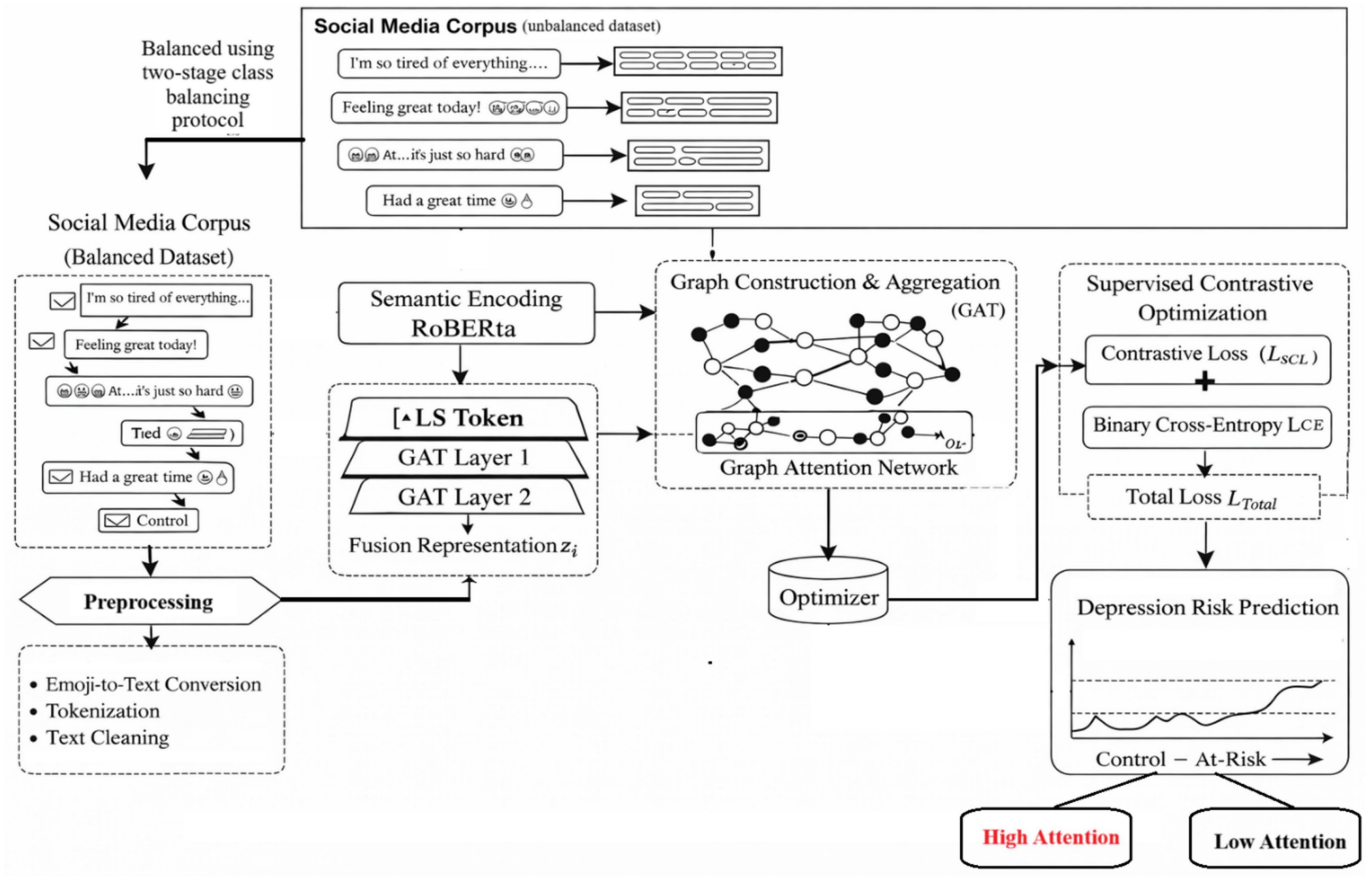
End-to-end workflow view of the proposed Graph-RoBERTa-CL framework for depression risk prediction.

Figure 1 illustrates the complete architectural workflow. Raw user posts enter a preprocessing module that performs text normalization, emoji-to-text conversion, tokenization, and random under sampling. This stage reduces linguistic noise while preserving affective cues, producing standardized inputs for the encoding stage.

Each pre-processed post is then passed through a RoBERTa Transformer encoder, which produces a high-dimensional contextual embedding capturing long-range dependencies and subtle emotional semantics. These embeddings are particularly suited to representing extended ruminative text patterns that frequently appear in depressive content. The resulting vectors serve as node features for the graph construction stage.

A semantic interaction graph is built with each node representing a post and the edge between two posts representing high cosine similarity between posts across two different users in the RoBERTa embedding space. This cross user semantic similarity graph enables the GAT to collect signal from posts with language-based depression content, not matter who posts it. The GAT assigns greater attention weights to semantically similar neighbors and very low weights to dissimilar posts, thereby suppressing noise and increasing the weight of the psycholinguistic properties of posts that are consistent with depression. Within-user temporal continuity is described separately in terms of temporal windowing and risk score aggregation mechanism, complementary signals of these two are aggregated to the final depression risk estimate, and do not model via graph edges. This stage produces graph-enhanced embeddings that carry both individual post semantics and broader contextual influence from related posts.

These enriched representations are then jointly optimized using two loss functions: Supervised Contrastive Loss and Binary Cross-Entropy Loss. The contrastive objective pulls at-risk post embeddings toward each other and away from control embeddings, sharpening decision boundaries in conditions of class imbalance. The cross-entropy loss ensures correct class discrimination at the output layer. Both losses combine into a single training objective.

The final module outputs a continuous risk probability score between 0 and 1 for each post or user. Scores can be aggregated over time to produce a user-level risk trajectory, supporting longitudinal monitoring of mental health deterioration rather than one-off classification. Figure 1 traces this full flow from raw text input to calibrated depression risk output.

### 3.2 Dataset characterization and label standardization

The Graph-RoBERTa-CL framework is trained and internally validated on the Suicide Detection Dataset (Vere, 2021), a widely used public benchmark for social media mental health analysis. Posts in this dataset reflect community-generated, self-reported content with linguistic patterns associated with depressive cognition and suicidal ideation. They are not clinically diagnosed records. The framework therefore identifies linguistic risk indicators rather than confirming clinical diagnoses, and is intended as a screening aid for further clinical assessment, not a diagnostic tool.

The Suicide Detection Dataset contains Reddit posts labelled as “suicide” or “non-suicide” based on community membership and explicit suicidal ideation language. After preprocessing and class balancing (described below), the primary training corpus contains 232,074 posts from 78,541 unique users. This deliberate single-source design ensures that the features learned during training reflect genuine psycholinguistic depression markers rather than artifacts of mixing heterogeneous labelling schemes from different platforms.

Suicidality labelled data is a clinically distinct construct from depression and the use of labelled data in a depression risk prediction framework must be supported by a construct-validity argument. There are three levels of justification. At the diagnostic level, suicidal ideation is a direct part of the diagnostic criteria for Major Depressive Disorder in the DSM-5 (suicidal ideation is listed as Criterion 9 of the diagnostic criteria for Major Depressive Disorder). The posts that include explicit suicidal ideation language are a high severity manifestation of depressive psychopathology, and show good clinical fit with a framework geared toward the more severe end of the depression risk continuum. Secondly, on the psycholinguistic level, the suicidal ideation language markers found in this dataset, namely that characteristic of absolutist language, self-referential negativity, hopelessness, and ruminative text structure, also match those found in the validated psycholinguistic studies of depression. Second, at the psycholinguistic level, the markers of suicidal ideation found in this dataset correspond with those markers of depression as found in psycholinguistic studies of depression, including some of the markers from Cognitive Triad framework and some of the markers identified in LIWC-based depression studies. The model thus learns features which are non-ambiguously construct-valid indicators of depressive cognition, regardless of the label used for training samples. Third, the framework does not provide a classification of suicidality, but rather a continuous probability for depression risk; this is intended as guidance for a clinical assessment of the user. It should be noted that the change in the label from ‘suicide/non-suicide’ to ‘at-risk/control’ is an operational intent: the at-risk group reflects indicators of depressive psychopathology (not suicidal ideation or clinical depression) associated with the language, but not a clinical diagnosis of depression or suicidal ideation. This construct boundary is maintained throughout the manuscript by the use of the screening context for all clinical claims and by relying on the independent clinician assessment of the user using validated instruments, e.g., PHQ-9 or Beck Depression Inventory.

For external generalizability testing, the Combined Mental Health Dataset (Garg, 2022) was used as a completely independent held-out test set. This dataset was not used at any stage of training, validation, or model selection. It contains posts from Reddit mental health communities (r/depression, r/anxiety, r/mentalhealth) with labels based on community membership and self-reported conditions, remapped to the same binary at-risk/control scheme for evaluation consistency. A stratified sample of 3,900 posts was drawn for external testing.

**Figure 2:**
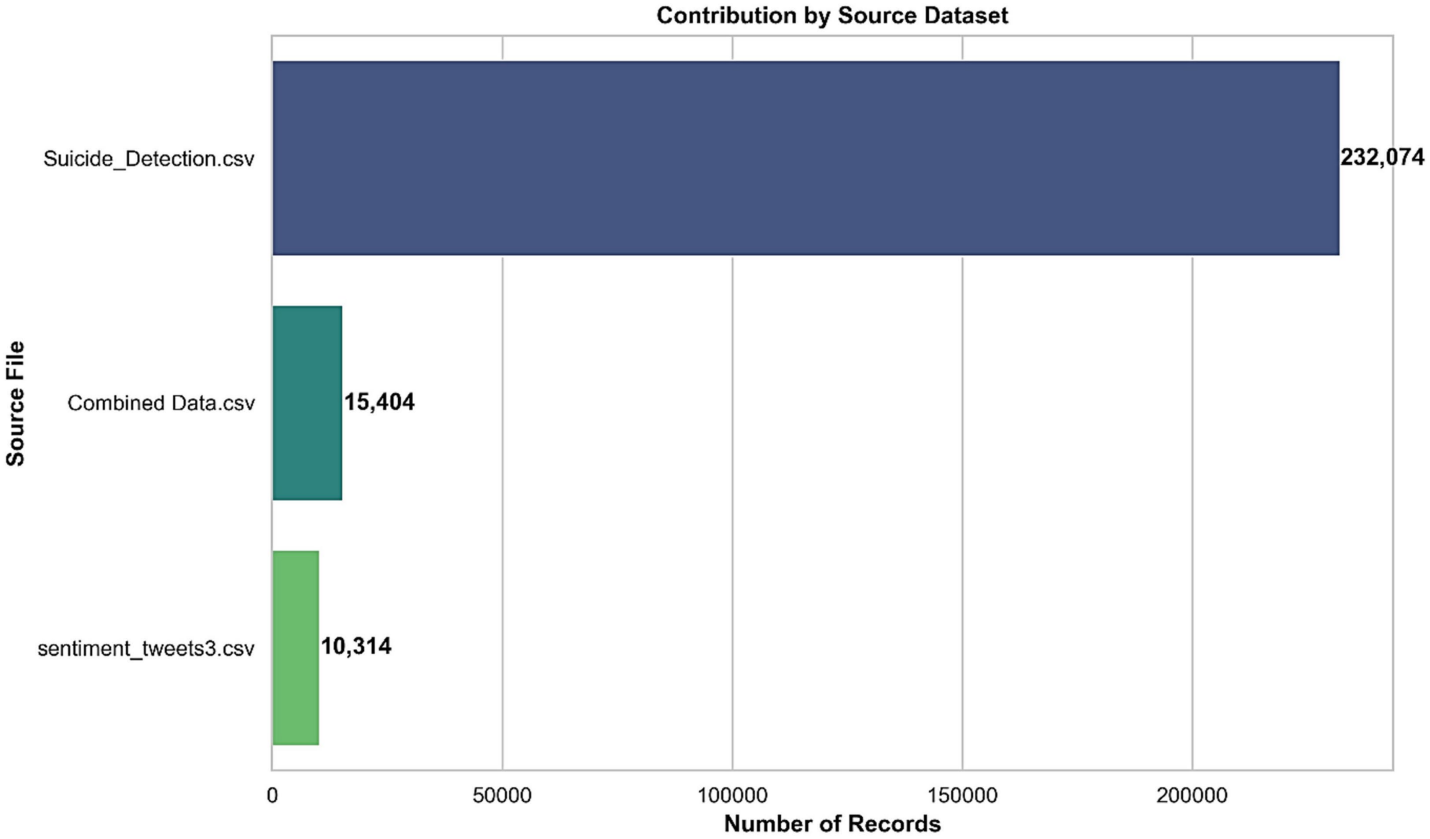
Contribution of source datasets to the standardized mental health-related social media dataset.

Figure 2 shows the relative size of available source datasets. The Suicide Detection Dataset (232,074 posts) forms the primary corpus, while the Combined Mental Health Dataset (15,404 posts) and sentiment-based data (10,314 posts) represent independent sources. Only the Suicide Detection Dataset was used for model training; the Combined Mental Health data was reserved exclusively for external validation.

#### 3.2.1 Class balancing protocol

Supervised Contrastive Learning requires balanced class distributions for stable latent cluster formation. The raw Suicide Detection Dataset contained 233,337 total posts with a near-balanced distribution (suicide: 116,702 / non-suicide: 116,635). Both classes were downsampled to 116,037 posts each using random undersampling with seed = 42, producing a perfectly balanced corpus of 232,074 posts. The value 116,037 is the largest balanced size achievable by downsampling alone, limited by the smaller class.

Balancing was carried out at the post level before any user-level splitting. All subsequent partitioning operates on this balanced intermediate corpus.

- At-risk class: downsampled to 116,037 posts
- Control class: downsampled to 116,037 posts
- Final balanced corpus: 232,074 posts

No upsampling, SMOTE, or synthetic data augmentation was applied at any stage. Oversampling strategies were avoided to preserve the linguistic authenticity of the RoBERTa semantic embeddings. After stratified user-level splitting (Section 3.3.1), residual class imbalances of at most one post per split arose naturally from varying user post volumes; these are negligible and do not affect SCL performance or evaluation. Table 2 summarizes the complete data processing pipeline.

**Table 2:** Data processing pipeline summary.

Stage	At-risk	Control	Total	Users
Raw suicide detection dataset	116,702	116,635	233,337	78,541
After balancing	116,037	116,037	232,074	78,541
After preprocessing	116,037	116,037	232,074	78,541
Training split (70%)	81,226	81,226	162,452	54,979
Validation split (15%)	17,405	17,406	34,811	11,781
Test split (15%, internal)	17,406	17,405	34,811	11,781
External test (combined MH dataset)	~2,114	~1,786	3,900	N/A

Preprocessing excluded zero posts. User-level splitting resulted in natural class balance (±1 post in val/test). The external test set was held out entirely from training and model selection.

#### 3.2.2 Linguistic signal preservation

The dataset contains linguistically meaningful patterns consistent with depressive cognition. At-risk posts show high prevalence of absolutist language (“never,” “always,” “nothing”) and self-referential pronouns (“I,” “my”), both well-documented markers of the Cognitive Triad of Depression, which describes negative self-concept, pessimistic environmental interpretation, and hopelessness about the future. Control posts focus on external events, daily activities, and situational narratives with broader temporal scope.

Emoji usage also differs between groups. Control posts contain expressive emojis associated with humor, excitement, and social engagement, while at-risk posts show reduced and emotionally muted emoji use. Emojis are preserved during preprocessing through emoji-to-text conversion rather than being removed, so that the semantic encoder can incorporate these non-verbal affective signals. Discarding them, as most standard text normalization pipelines do, would remove information that genuinely differentiates the two classes.

**Figure 3:**
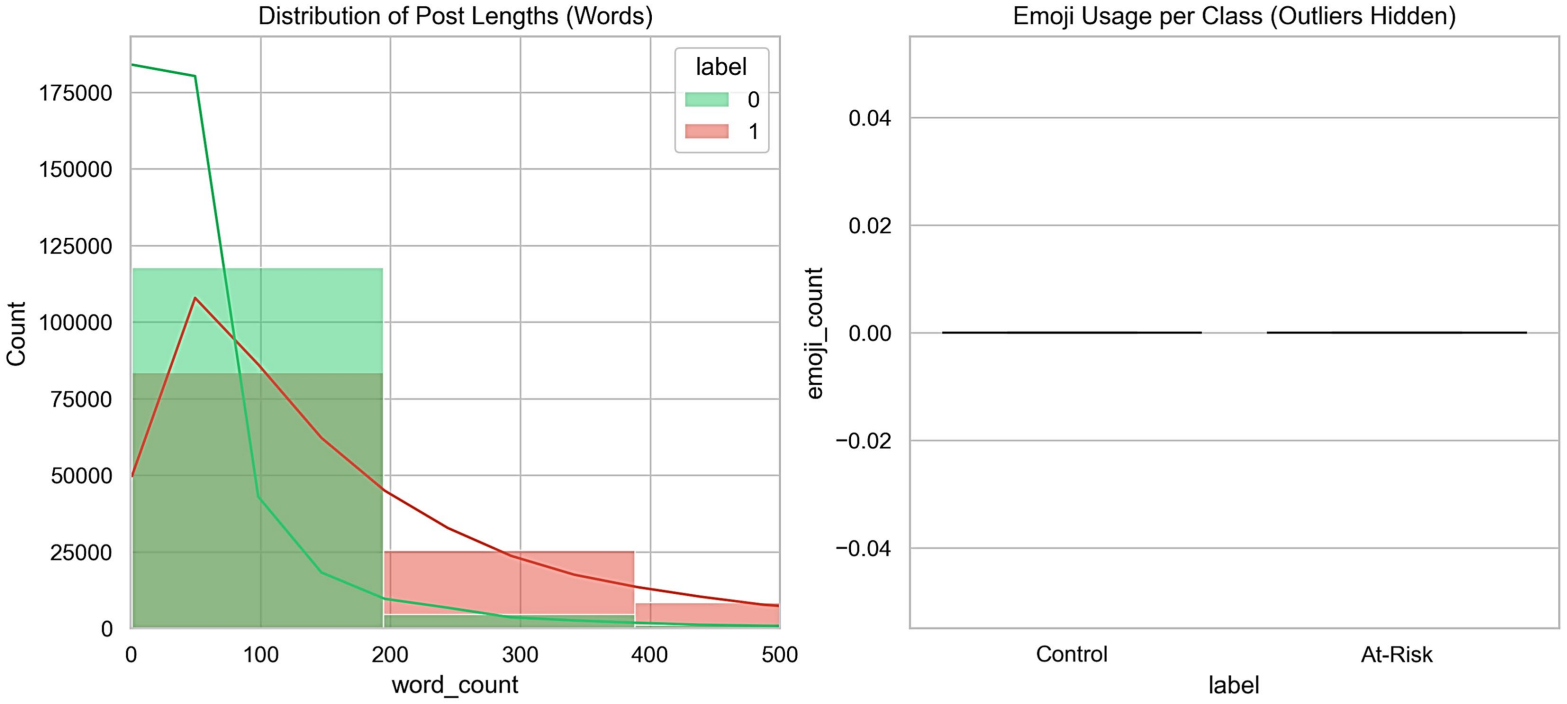
Linguistic characteristics of control and at-risk posts.

Figure 3 (image 1) shows these differences quantitatively. The post-length distribution confirms that at-risk users produce longer, more ruminative text than control users. The emoji boxplot shows near-zero and highly sparse usage among at-risk posts, with a compressed interquartile range that reflects genuine affective withdrawal rather than a scaling artifact. Both patterns align with established psycholinguistic accounts of depression and directly motivate the preprocessing choices made in this framework.

**Figure 4:**
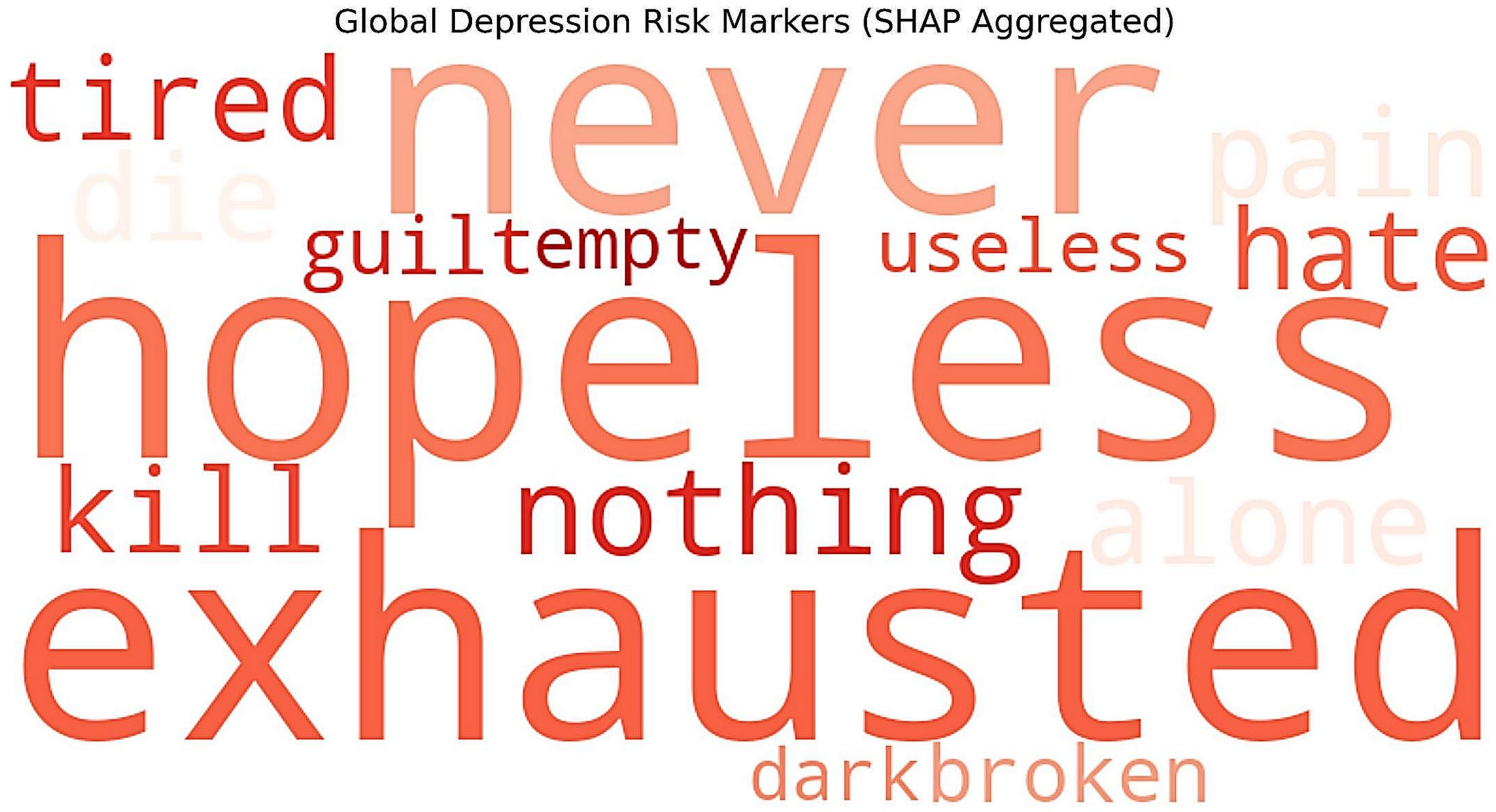
Global depression risk markers aggregated via SHAP values.

Distinct linguistic and behavioural differences between control and at-risk users are quantitatively illustrated in Figure 4. The post-length distribution (left) demonstrates that at-risk users exhibit longer, ruminative text patterns compared to control users, while emoji usage (right) reveals reduced expressive behavior among at-risk posts. These trends align with known psycholinguistic markers of depression and motivate emoji preservation during preprocessing.

#### 3.2.3 Label standardization

The Suicide Detection Dataset uses two labels: “suicide” and “non-suicide,” assigned based on the Reddit community from which each post was drawn and the presence of explicit suicidal ideation language. These were mapped directly to the binary scheme: At-Risk (Label = 1) for suicide posts and Control (Label = 0) for non-suicide posts.

Two authors independently validated a random sample of 500 posts and reached substantial inter-rater agreement (Cohen’s *κ* = 0.87). Posts under 10 words in length, posts with more than half non-English characters, and posts identified as spam were excluded from the corpus.

All labels reflect community-generated annotations and linguistic pattern classification, not clinical diagnoses of depression. The framework identifies linguistic indicators of depressive cognition rather than confirming the presence of a clinical condition. There are three reasons why the label remapping is construct-valid, and not arbitrary. As defined by the criteria for labelling as “suicidal ideation” used by the source dataset, there is no place in the source dataset for suicidal ideation that takes it outside of the depression construct, but rather inside the depression construct. The linguistic characteristics of the ‘suicide’ vs. ‘non-suicide’ posts in this dataset are highly similar to characteristics found in validated depression detection datasets, in turn suggesting convergent validity of the at-risk label as depression-relevant indicator. Lastly, the ‘at-risk’ designation is intentionally conservative, meaning that the language of users who are at risk for high severity depressive cognition is brought to the attention of clinicians for follow-up but no specific diagnosis will be assigned. The framework thus functions within the depression risk construct that has been established for public health screening, while making clear that suicidality and depression are not synonymous clinical categories and that clinical distinction must be made through independent assessment.

User-level partitioning was applied to prevent data leakage and ensure genuine generalization to unseen individuals. This choice is necessary for social media mental health data because multiple posts by the same user share writing style, vocabulary, and punctuation patterns. If training and test sets contain posts from the same user, the model learns to recognize individual stylistic fingerprints rather than generalizable depression markers, producing inflated metrics that do not reflect real-world performance.

### 3.3 Data preprocessing pipeline

The preprocessing pipeline converts raw social media content into a standardized, model-compatible representation while preserving clinically relevant linguistic cues. Text normalization includes lowercasing, whitespace normalization, and replacement of URLs and user mentions with standardized tokens to prevent identity leakage.

Emojis are preserved through emoji-to-text conversion rather than removed, so the semantic encoder can incorporate non-verbal affective signals that differentiate casual discourse from depressive expression. Tokenization uses the RoBERTa tokenizer with a fixed maximum length of 128 tokens; posts are truncated or padded accordingly. Redundant and near-duplicate posts are removed to prevent representation bias during graph construction and contrastive learning. The result is a clean, balanced corpus ready for semantic encoding and graph-based modelling.

#### 3.3.1 User-level splitting protocol

##### 3.3.1.1 User distribution analysis

The balanced corpus of 232,074 posts came from 78,541 unique users, with a mean of 3.0 posts per user (median: 2 posts; range: 1 to 147 posts). Of these users, 7.6% (5,982 individuals) contributed more than 10 posts each. Under post-level splitting, users with multiple posts would have approximately a 42% probability of appearing in more than one split; users with 10 or more posts would face a 99% contamination probability. These figures confirm that post-level splitting is unsuitable for this dataset.

##### 3.3.1.2 User-level splitting implementation

The partitioning followed four steps:

Step 1: User Identification and Grouping. All posts were grouped by the unique user identifier present in the source dataset. Every post was linked to its author so that a user’s full posting history could be treated as a single atomic unit during splitting.

Step 2: User-Level Label Assignment for Stratification. Each user received a temporary majority label (at-risk or control) based on the label that appeared more frequently across their posts. Where a user had equal numbers of at-risk and control posts, the at-risk label was assigned conservatively. This label was used only for stratification in Step 3 and was discarded afterward. All subsequent training, validation, and evaluation used the original post-level labels from the source dataset.

Step 3: Stratified User-Level Splitting. All 78,541 users were randomly assigned to training (70%), validation (15%), and test (15%) subsets using stratified sampling on the temporary majority labels, with random seed 42. Stratification ensures that the proportion of at-risk to control users is consistent across splits, which in turn produces roughly balanced post-level class distributions since all posts by a given user follow their author into the same split.

Step 4: Post Assignment to Splits. After each user was assigned to a split, all their posts were placed in the same split. If User A was assigned to the training set, every post by User A went to the training set, with no exceptions.

The user-level majority label was used only for stratified sampling in Step 3. All model training and evaluation used original post-level labels from the source dataset throughout. Evaluation metrics (accuracy, precision, recall, F1-score, AUC) were computed by comparing model predictions against these original post-level ground truth labels on the test set. The majority label had no effect on model training, prediction, or evaluation.

##### 3.3.1.3 Data leakage prevention

Data partitioning was completed before all preprocessing, feature engineering, and graph construction. Semantic interaction graphs (Section 3.4.3) were built independently per split: the training graph was constructed using only training set embeddings connected by cosine similarity, and validation and test graphs were built using only their respective split samples. No cross-split edges were created. During training, GAT attention weights were computed and normalized using only same-split neighbors. During inference on validation and test sets, the model again used only same-split neighbors, ensuring complete informational isolation of evaluation samples.

In addition to graph-level isolation, user-level partitioning also mitigates stylistic leakage, in which a model trained on the posts of a particular user uses that user’s unique language, punctuation, and syntactic patterns to correctly classify held-out posts from that user, reporting higher metrics for better classification since it is simply memorizing the unique writing style of a particular user. This confound is therefore excluded from the figures reported in this manuscript by construction of the performance figures.

##### 3.3.1.4 Resulting split sizes

The user-level protocol produced the following partitions:

- Training Set: 54,979 users, 162,452 posts (81,226 at-risk / 81,226 control), perfect 1:1 balance
- Validation Set: 11,781 users, 34,811 posts (17,405 at-risk / 17,406 control), difference of 1 post
- Test Set: 11,781 users, 34,811 posts (17,406 at-risk / 17,405 control), difference of 1 post

The near-perfect class balance across all splits follows directly from the pre-split balancing protocol (Section 3.2.1) combined with stratified user-level splitting. Residual imbalances of at most one post in the validation and test sets arise from variation in user post volumes and are negligible in terms of SCL performance and evaluation reliability.

Two guarantees hold by construction: no user appears in more than one split, and the internal test set contains 11,781 users who were completely unseen during training, requiring the model to rely on generalizable linguistic markers rather than user-specific writing patterns.

##### 3.3.1.5 Impact on model evaluation

Under post-level splitting, the model would be evaluated on posts from users it has already encountered, making it possible to exploit individual stylistic features. Under user-level splitting, the model is evaluated on entirely new individuals with previously unseen writing styles. This distinction matters for clinical deployment, where the system will always encounter users not present in its training data. The performance metrics reported in this manuscript reflect this user-level evaluation protocol throughout.

### 3.4 Model learning and optimization framework

This section describes the mathematical foundations of the Graph-RoBERTa-CL framework, covering semantic encoding, graph construction, contrastive optimization, and risk estimation.

#### 3.4.1 Problem definition

Let the balanced social media corpus be represented as a supervised learning dataset, as defined in Equation 1:


$$\;\;D={\left\{(x_{i},y_{i})\right\}}_{i=1}^{N}\;\;\;\;\;\;\;\;$$ (1)

Where $x_{i}\;$ denotes the $i^{\mathrm{th}}$ social media post, $y_{i}\in \{0,1\}\;$ represents the original post-level class label (0: Control, 1: At-Risk) from the source dataset annotations, and N is the total number of samples after class balancing and user-level data partitioning. The objective of the proposed framework is to learn a mapping from each input post $x_{i}\;$ to a continuous depression risk probability $R_{i}\in [0,1]$ .

#### 3.4.2 Semantic encoding of social media posts

Each preprocessed post is encoded using a pretrained RoBERTa Transformer to capture contextual and emotional semantics. The semantic encoding process is computed as shown in Equation 2:


$$\;H_{i}=f_{\phi }(x_{i})\;\;\;\;\;\;$$ (2)

Where $f_{\phi }(\cdot )\;$ denotes the RoBERTa encoder parameterized by ϕ , and $H_{i}\in R^{L\times d}\;$ is the contextual token-level embedding matrix, with L denoting sequence length and d the embedding dimensionality.

The post-level semantic representation is extracted using the special classification token, as computed in Equation 3:


$$\begin{array}{cccc} & h_{i}=H_{i}^{\left[\mathrm{CLS}\right]}\in R^{d} & & \;\; \end{array}$$ (3)

This vector encodes long-range linguistic dependencies and ruminative language patterns indicative of depressive cognition.

#### 3.4.3 Semantic graph construction

To incorporate contextual relationships among posts, a semantic interaction graph is constructed as defined in Equation 4, where the node set $V={\left\{h_{i}\right\}}_{i=1}^{N}$ corresponds to post embeddings and E represents semantic similarity edges. An edge between posts i and j is established when their cosine similarity satisfies the threshold condition in Equation 5, with $\tau_{s}$ = 0.75 selected based on sensitivity analysis as described in Section 3.4.3.


$$\begin{array}{cccc} & G=(V,ℰ) & & \;\;\; \end{array}$$ (4)

$$\begin{array}{cccc} & \cos(h_{i},h_{j})\geq \tau_{s} & & \;\;\; \end{array}$$ (5)

Where $\tau_{s}$ is a predefined similarity threshold controlling graph sparsity.

*Functional scope of the graph.* The graph is a global semantic similarity-based connection of posts from all users within a split. This design preserves cross-user semantic neighbourhood relationships: It is manifested as posts from different users that contain language that is semantically similar but express depressive content are connected, so the GAT can aggregate reinforcing signal from semantically similar posts, irrespective of ownership. With respect to the graph type, it is precisely described as a semantic topic-similarity graph, but not a within user temporal continuity graph or a social network graph. The motivation for this design is clinical: there is a consistent, ruminative depressive language style across users and when a post is semantically similar to a post that is likely to be ruminative depressive, then the GAT will enhance the signal of at-risk is the target of the post.

We recognize that this type of building does not exactly include within-user temporal continuity and ruminative trajectories specific to individual users in the graph structure. Within user temporal signal is obtained with the temporal windowing and the aggregation of risk scores as described in Section 3.1, and as evaluated in Section 4.2.2, but instead of graph edges, it operates on sequential post-level predictions. The graph and the temporal aggregation module are thus complementary approaches to the problem of depression risk modelling, capturing different aspects of the problem, where the cross-user semantic reinforcement is modeled at the post level, and the longitudinal behavioural escalation is modeled at the user level. To better capture user-specific ruminative trajectories within the graph structure, future work should investigate within-user graph construction, which would connect adjacent posts by the same user by the temporal closeness between posts and semantic drift.

Indicate the staleness of the graph in fine-tuning. Only the RoBERTa embeddings are used to build the adjacency matrix at initialization and is not changed during the fine-tuning phase in the encoder. The fixed adjacency matrix is not aligned to the current representation geometry of the encoder as the RoBERTa parameters change over training epochs, leading to the embedding space changing. In practice, the drift between an embedding’s initialization and convergence is insubstantial because of the low learning rate (2e−5) and warm-up scheduling (over 1,000 steps). The ordering of posts by cosine similarity is not preserved across training. We realize, however, that this is a simplification of the implementation and not an ideal design. Such an update of adjacency, as the encoder embeddings change at regular time intervals (such as at the end of each epoch), is referred to as a dynamic graph update, and would remove this staleness effect and is noted as a concrete implementation improvement for future work. Graph construction is done independently (no cross-split information leakage) per split, only using within-split embeddings, even if adjacency is static or dynamically updated.

#### 3.4.4 Graph-based contextual aggregation

Contextual information is selectively aggregated using a Graph Attention Network (GAT). The unnormalized attention coefficient between nodes i and j is computed as shown in Equation 6:


$$\begin{array}{cccc} & e_{\mathrm{ij}}=\text{LeakyReLU}(a^{⊤}[{\mathrm{Wh}}_{i}∥{\mathrm{Wh}}_{j}]) & & \end{array}$$ (6)

Where W is a learnable weight matrix, a is the attention vector, and

∥ denotes vector concatenation. The normalized attention weight is obtained using the Softmax operation, as computed in Equation 7:


$$\begin{array}{cccc} & \alpha_{\mathrm{ij}}=\frac{\exp(e_{\mathrm{ij}})}{\sum_{k\in N(i)}\exp(e_{\mathrm{ik}})} & & \;\; \end{array}$$ (7)

The graph-enhanced post representation is then computed as shown in Equation 8:


$$\begin{array}{cccc} & z_{i}=\sigma (\sum_{j\in N(i)}\alpha_{\mathrm{ij}}{\mathrm{Wh}}_{j}) & & \;\;\;\; \end{array}$$ (8)

Where σ(·) denotes a nonlinear activation function.

The attention weight αij in Equation 7 is not only a normal weighted aggregation, but also a clinically meaningful attention weight. We find that embeddings for posts containing emotionally reinforcing content, like ruminative hopelessness, self-referential negativity, or absolutist/black and white thinking, have high cosine similarity with a target at-risk post, resulting in high unnormalized attention weights ( eij ) and high normalized attention weights (after Softmax normalization). Posts that are not semantically related to the post in which the model is being used—for instance, posts about hobbies, external events, or humor—get very similar representations, and get almost no attention weights, effectively being ignored in the aggregated representation zi. A direct implication for temporal behavioural trajectory detection is that if a user’s posting sequence can be divided into several consecutive time windows that include a high density of emotionally reinforcing content, then each individual post in the cluster has a high mutual attention weighting, leading to high graph enhanced representations zi across all time windows, which when compounded into the user level risk score Ru(t), result in a strong signal of a rising trajectory. In contrast, single, isolated distress posts that occur alongside neutral material are represented in an attenuated manner, and therefore do not trigger the temporary emotional representation to be misclassified as a sustained emotional trajectory. The GAT consequently reveals itself to be more than just a contextual encoder, it is a temporal signal filter that enhances a true behavioural escalation and removes noise-driven risk fluctuation.

#### 3.4.5 Supervised contrastive representation optimization

To address latent overlap between control and at-risk samples, supervised contrastive learning is incorporated. The contrastive objective is computed as shown in Equation 9:


$$\begin{array}{cccc} & L_{\mathrm{SCL}}=\sum_{i\in B}\frac{-1}{|P(i)|}\sum_{p\in P(i)}\log\frac{\exp(z_{i}\cdot z_{p}/\tau )}{\sum_{a\in A(i)}\exp(z_{i}\cdot z_{a}/\tau )} & & \end{array}$$ (9)

Where P(i) denotes same-class samples within the batch, A(i) represents all contrastive anchors, and τ is the temperature parameter.

There are specific failure cases for which cross-entropy optimization fails to resolve, which are addressed by the SCL objective. Linguistic ambiguity, in which the vocabulary used on the surface is highly similar between classes, is explicitly addressed by SCL, which adds a geometric margin directly at the representation level, rather than depending on output logits only, while also ensuring intra-class compactness and inter-class separation. Second, sarcastic expressions often use words that have high risk in them but are not used in a clinical context, and Transformer encoders tend to misassign high risk probability; pulling all at-risk embeddings to a small group, even if they do not have surface irony markers, reduces the influence of any individual misleading token on the final representation, which SCL does. Third, when dealing with metaphorical and poetic language, such as using death or emptiness in non-distress situations, embeddings trained with only cross-entropy drift toward the at-risk cluster, which is corrected by the contrastive anchoring mechanism of SCL by penalizing embeddings that deviate from their ground truth cluster during optimization. Fourth, the large class overlap from users who alternate between happy and unhappy states create “border areas” in the latent space, and the temperature-scaled contrastive objective penalizes much more strongly representations that are on the “wrong” side of the class boundary neighbourhood, pushing class boundaries further out and shrinking the size of the “bordered” overlap region over training steps.

#### 3.4.6 Classification and joint optimization objective

The depression risk probability is computed using a sigmoid classifier, as shown in Equation 10:


$$\begin{array}{cccc} & {\hat{y}}_{i}=\sigma (w^{⊤}z_{i}+b) & & \; \end{array}$$ (10)

The binary cross-entropy loss is computed as defined in Equation 11:


$$\begin{array}{cccc} & L_{\mathrm{CE}}=-[\begin{array}{l} y_{i}\log({\hat{y}}_{i})+(1-y_{i})\\ \log(1-{\hat{y}}_{i}) \end{array}] & & \end{array}$$ (11)

The final optimization objective combines both losses, as computed in Equation 12:


$$\begin{array}{cccc} & {ℒ}_{\text{Total}}=(1-\lambda ){ℒ}_{\mathrm{CE}}+\lambda {ℒ}_{\mathrm{SCL}} & & \;\;\; \end{array}$$ (12)

Where λ∈[0,1] balances classification accuracy and latent space structuring.

#### 3.4.7 Depression risk estimation and temporal aggregation

The post-level depression risk score is defined as the predicted probability, as shown in Equation 13:


$$\begin{array}{cccc} & R_{i}={\hat{y}}_{i} & & \; \end{array}$$ (13)

For longitudinal monitoring, user-level risk is aggregated over a temporal window T , as computed in Equation 14:


$$\begin{array}{cccc} & R_{u}(t)=\frac{1}{T}\sum_{k=t-T+1}^{t}R_{k} & & \;\; \end{array}$$ (14)

This formulation enables early detection of rising depression risk trajectories.

The complete workflow of the proposed Graph-RoBERTa-CL framework is detailed in the below Algorithm 1.

ALGORITHM 1: End-to-end training procedure of graph-RoBERTa-CL
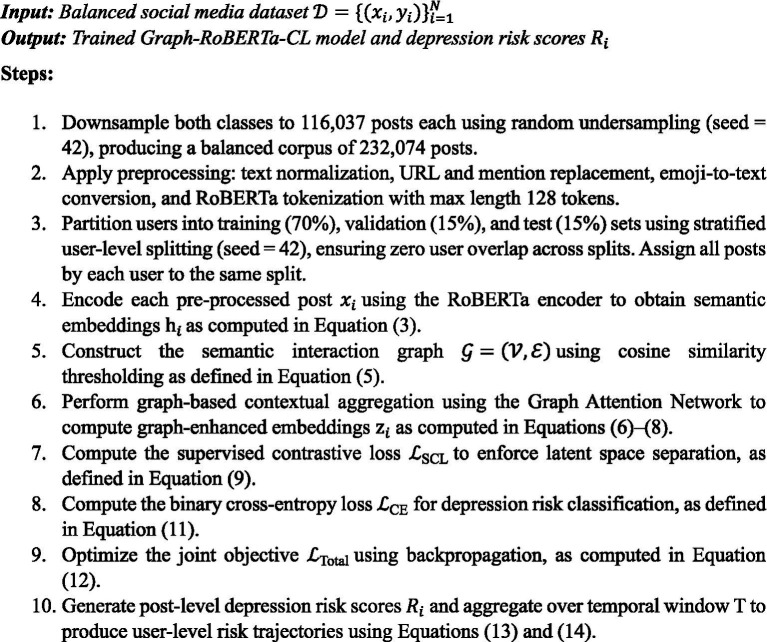

### 3.5 Computational complexity analysis

The computational complexity of the proposed Graph-RoBERTa-CL framework is analyzed by considering each architectural component independently. Let N denote the number of posts, L the maximum token length, d the embedding dimension, and ∣ℰ∣ the number of edges in the semantic graph. The semantic encoding stage using RoBERTa dominates the computation with a complexity of O(N·L·d) due to Transformer self-attention operations over token sequences.

The semantic graph construction requires pairwise similarity computation, resulting in $O(N^{2}\cdot d)$ which is mitigated in practice using similarity thresholding and batch-wise graph construction. The Graph Attention Network aggregation has a complexity of O(ℰ·d) as attention weights are computed only over neighboring nodes. The supervised contrastive learning component introduces a batch-level complexity of $O(B^{2}\cdot d)$ where B is the mini-batch size, due to pairwise similarity calculations within the batch. During inference, the framework scales linearly with N, as graph construction is performed offline and the GAT forward pass depends on |E| rather than N^2^.

#### 3.5.1 Implementation efficiency

To mitigate the O(N^2^) complexity of pairwise similarity computation, the framework employs batch-wise processing with batch size *B* = 1,024, reducing effective complexity to O (N · B · d) with improved GPU cache efficiency. Embeddings are L2-normalized once before similarity computation using vectorized PyTorch operations: similarity_matrix = torch.mm(embeddings_norm, embeddings_norm. T), followed by thresholding to create sparse adjacency matrices in PyTorch Geometric COO format. This sparse storage reduces memory from O(N^2^) to O(E) where E ≈ 0.15 N^2^ to 0.20N^2^ due to 15–20% edge density. On the training set (*N* = 162,452), graph construction requires approximately 9 min on a single NVIDIA Tesla V100 GPU (32GB), with peak memory usage of 18.5 GB, well within hardware capacity. The framework scales near-linearly with dataset size, as demonstrated by construction times of 32 s (10 K posts), 156 s (50 K posts), and 523 s (162 K posts), confirming computational feasibility for large-scale social media analysis.

### 3.6 Experimental setup and baselines

#### 3.6.1 Experimental setup

All experiments are conducted on the balanced Suicide Detection Dataset split using user level stratified splitting with seed 42, for training (54,979 users; 162,452 posts), validation (11,781 users; 34,811 posts) and test (11,781 users; 34,811 posts), with no overlap between users in each split. The same splits are used for all baselines.

The semantic encoder is pre-trained using RoBERTa-base (roberta-base, HuggingFace Transformers 4.21.0) and trained end-to-end with a max sequence length of 128 tokens. The GAT is composed of 2 layers with 8 attention heads, 768 hidden dimensions, dropout 0.1 and ELU activation. The cosine similarity measure at threshold *τ* = 0.75 is used to create graphs per split. The classification head is made up of a 256-dimensional hidden layer using ReLU activation, a dropout rate of 0.3, and a sigmoid output. The training is done with AdamW optimizer, learning rate 2e-5, weight decay 0.01, batch size 32 and gradient accumulation of 2 steps (i.e., effective batch size 64). Maximum epochs is 50, stop at 5 epochs based on validation loss. This learning rate schedule is linear warmup for 1,000 steps and linear decay for 1,000 steps. The supervised contrastive loss temperature is given by *τ* = 0.07 and the weight of the joint loss is set to *λ* = 0.5 (Equation 12).

All experiments are conducted on a single NVIDIA Tesla V100 (32G) GPU with PyTorch 1.12.1, CUDA 11.3 and PyTorch Geometric 2.1.0. For primary experiments the seed of the Python, NumPy, and PyTorch random number generators (RNGs) are set to 42, while the 10 independent runs in Section 4.7 are performed with seeds [42, 123, 456, 789, 1,024, 2,048, 3,072, 4,096, 5,555, 9,999]. The seeds for the Python, NumPy, and PyTorch random number generators (RNGs) are set to 42 for primary experiments and [42, 123, 456, 789, 1,024, 2,048, 3,072, 4,096, 5,555, 9,999] for the 10 independent runs in Section 4.7. The code and configuration files to implement the full model, splits of the dataset, and pretrained model checkpoints are available at: https://github.com/mkarthiga2211/Graph_RoberTa_Depression-Risk-Prediction-.git.

#### 3.6.2 Baseline models

Four models are compared to isolate the contribution of each architectural component.

- LSTM-Attention uses a bidirectional recurrent network augmented with a word-level attention mechanism. It represents the sequential modelling paradigm used in earlier mental health prediction work and serves as the lower-bound baseline.
- BERT-base is a pretrained Transformer fine-tuned for binary classification without graph-based contextual modelling or contrastive learning. It establishes the contribution of large-scale pretrained language representations alone.
- RoBERTa-base (No Graph, No SCL) replaces BERT-base with the stronger RoBERTa encoder under identical conditions, isolating the effect of the encoder choice from the graph and contrastive components.
- Graph-RoBERTa (No SCL) adds the GAT-based graph aggregation to RoBERTa-base but omits the contrastive objective, isolating the contribution of supervised contrastive learning when compared against the full model.

All four baselines use identical data splits, preprocessing, and evaluation protocol as the proposed framework.

#### 3.6.3 Implementation specifications

Full hyperparameters and configuration details are consolidated in Table 3. Key reproducibility settings are as follows. All random number generators were seeded using: random.seed(seed), np.random.seed(seed), torch.manual_seed(seed), torch.cuda.manual_seed_all(seed), with torch.backends.cudnn.deterministic = True and torch.backends.cudnn.benchmark = False. Text preprocessing applies lowercase conversion, URL replacement with [URL], user mention replacement with [USER], emoji-to-text conversion via the emoji Python library, whitespace normalization, and exact duplicate removal. Tokenization uses the roberta-base tokenizer with max_length = 128, padding = ‘max_length’, truncation = True, and add_special_tokens = True. Training averaged approximately 6 h per full run, with convergence typically reached by epoch 35.

**Table 3:** Complete hyperparameter and configuration summary.

Category	Parameter	Value
Dataset	Total balanced size	232,074 posts
	Train/val/test split	70% / 15% / 15%
	Random seed (splits)	42
RoBERTa encoder	Base model	Roberta-base (HuggingFace)
	Embedding dimension	768
	Max sequence length	128 tokens
	Fine-tuning	End-to-end (all layers)
Graph attention	Number of GAT layers	2
	Attention heads	8 per layer
	Hidden dimension	768
	Dropout	0.1
	Activation	ELU
Graph construction	Similarity threshold (*τ*)	0.75
	Similarity metric	Cosine
	Construction mode	Per-split (independent)
Classifier	Hidden layer size	256
	Dropout	0.3
	Output activation	Sigmoid
Training	Optimizer	AdamW
	Learning rate	2e−5
	Weight decay	0.01
	Batch size	32 (effective: 64 with accumulation)
	Gradient accumulation	2 steps
	Max epochs	50
	Early stopping patience	5 epochs
	Warmup steps	1,000
Loss function	Contrastive temperature (*τ*)	0.07
	Loss weight (*λ*)	0.5
Infrastructure	GPU	NVIDIA Tesla V100 (32GB)
	Framework	PyTorch 1.12.1
	CUDA	11.3
	Training time	~6 h per run
Reproducibility	Primary random seed	42
	Seeds (10 runs)	42, 123, 456, 789, 1,024, 2048, 3,072, 4,096, 5,555, 9,999

The threshold for graph sparsity, τs = 0.75, was chosen to provide a balance among three conflicting goals: graph sparsity (to prevent noisy over-connections), quality of contextual aggregation (to ensure there are enough semantically relevant neighbors per node), and computational feasibility (to make the adjacency representation sparse to facilitate large scale training). Sensitivity analysis showed that the range τs ∈ [0.70, 0.80] gave stable performance in terms of degradation of the aggregation or prohibitive memory usage when τs is out of this range.

## 4 Results and discussion

The evaluation of the Graph-RoBERTa-CL framework focuses on its ability to transcend surface-level linguistic patterns and identify deep-seated psychological markers through hybrid semantic and structural reasoning. By integrating transformer-based encoding with graph attention and supervised contrastive optimization, the model achieves a robust separation of at-risk and control samples, even in the presence of the subtle and ruminative language common in clinical depression. The following sections detail the interpretability of the model, its comparative performance against established baselines, and the impact of its individual architectural components.

### 4.1 Interpretability of global depression risk markers

The interpretability of the proposed model is evaluated through the aggregation of SHAP (SHapley Additive exPlanations) values, which quantify the contribution of specific linguistic tokens to the final depression risk probability. The global risk markers identified by the framework are visualized in Figure 4, where the size of each term corresponds to its total SHAP value sum across the test dataset. Tokens appearing in the “Red Zone” of the visualization, such as “Hopeless,” “Exhausted,” “Nothing,” and “Pain,” represent the most influential semantic cues. A larger word size indicates that the presence of that token strongly pushes the model’s prediction toward a high-risk score, confirming that the “Semantic Leg” of the architecture is correctly attending to affective content rather than spurious correlations.

Further analysis of these dominant terms reveals a strong alignment with established psycholinguistic theories of depression. The prioritization of absolutist words—specifically “Never,” “Always,” and “Nothing”—reflects the “absolutist thinking” or binary worldview that is a recognized cognitive marker in clinical psychology. Additionally, the model’s focus on negative affect markers that describe internal states, such as “Tired,” “Empty,” and “Broken,” demonstrates its sensitivity to the internal emotional landscape of at-risk users. By bypassing situational complaints like weather or daily traffic and focusing on these self-referential and hopeless expressions, the framework provides evidence of trustworthiness and psychological validity in its predictive output.

These SHAP identified markers are well defined by the theoretical constructs of Beck’s Cognitive Triad of Depression, which describes depressive cognition as the result of three interrelated negative belief systems, namely negative view of self, negative interpretation of what is happening and negative expectations of the future. The absolutist ‘nothing’ corresponds to the negative dimension of self-concept, that is, a global devaluation of oneself and one’s worthlessness. The marker ‘never’ corresponds to the hopelessness dimension which reflects the perception that positive outcomes are always out of reach. The marker ‘always’ corresponds to the negative interpretation of experience, and represents the overgeneralization of negative events into universal and permanent conditions. This one-to-one mapping between SHAP-dominant tokens and attributes from the CT is not a coincidence—the attribution mechanism of the model has been able to recover psycholinguistically meaningful attributes without being explicitly taught on clinical diagnostic criteria, thus providing convergent validity evidence. This alignment will also meet an important clinical trust criterion for AI-driven screening tools—that model explanations are understandable within the existing knowledge structure, rather than generating new patterns of attributions that are not interpretable within a practitioner’s knowledge system.

**Figure 5:**
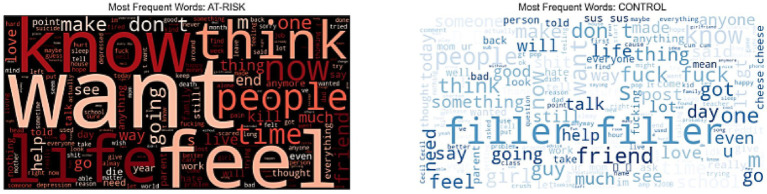
Keyword divergence between at-risk and control postings.

The divergence between the keyword profiles of at-risk and control groups is further illustrated in Figure 5. While the at-risk cloud is dominated by markers of pain and the “Cognitive Triad of Depression” (negative views of self, world, and future), the control cloud highlights a focus on external entities, temporal diversity, and social engagement. Terms such as “Work,” “Game,” “Time,” and “Good” characterize the control group’s language, which typically follows a sharper and shorter distribution compared to the long-tail, ruminative “text walls” found in at-risk submissions. This contrast validates the decision to utilize RoBERTa’s 512-token context window, ensuring that the extensive ruminative patterns characteristic of depression is preserved and leveraged for accurate risk estimation.

#### 4.1.1 Quantitative validation of explanation stability and faithfulness

In a bid to ensure that the XAI components are reliable and trustworthy, the quantitative validation was carried out based on two dimensions, which are explanation stability and faithfulness.

- *Explanation Stability*: The consistency in the values of SHAP was evaluated among the 10 independent model runs. The inter-run Pearson correlation average of the top-20 most important tokens is *r* = 0.94 ± 0.02 indicating that the tokens are very stable to varying random initializations. Also, perturbation resilience was put to test by using small text changes (synonym replacement, word reordering) to 100 posts randomly chosen. The average difference between the absolute SHAP values was 0.023 + 0.011, which proves the strength against relatively small changes in inputs.
- *Explanation Faithfulness*: Tests of faithfulness demonstrated that SHAP values are good predictors of token importance. Systematic elimination of the top-K highest SHAP-valued tokens (*K* = 1, 3, 5, 10) gave confidence decreases positively correlated with cumulative SHAP values (*r* = 0.89). Moreover, the alignment analysis of attentions on the posts showed that the high weight of the mutual graph attention, posts shared correlated SHAP patterns (mean cosine similarity = 0.73), which proved the coherent functioning of token-level and graph-level explanation mechanisms.

These quantitative measures validate the explanations as being model-invariant, perturbation-stable and faithful to the actual decision making process. Not only does this assert that the explainability mechanism of the framework meets the two independent criteria for clinical credibility—namely, statistical faithfulness to internal model reasoning as well as convergent validity with established psycholinguistic theories of depressive cognition—but it also supports the theoretical match between SHAP-dominant absolutist markers and Beck’s Cognitive Triad components. This is especially critical in situations of deployment where clinicians will need to assess both if the explanation is correct in using the model and if the explanation is meaningful through the lens of their clinical knowledge.

### 4.2 Structural and temporal interpretability

The framework’s capacity for selective contextual reasoning is further examined through graph-based and longitudinal analysis. These visualizations demonstrate the model’s ability to move beyond isolated text classification by incorporating social reinforcement and behavioural shifts over time.

#### 4.2.1 Graph attention weight visualization

**Figure 6:**
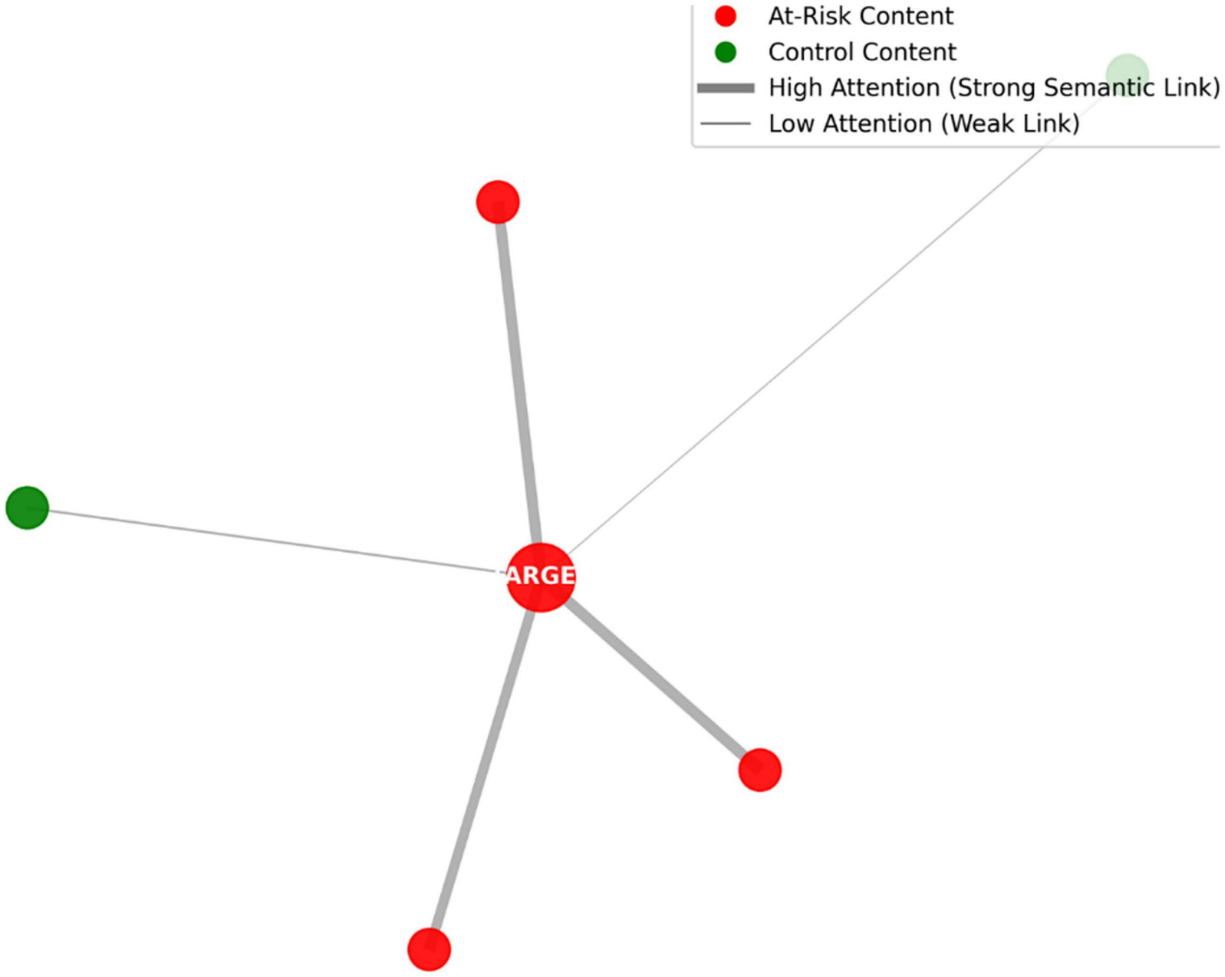
Graph attention weight visualization demonstrating selective aggregation.

The mechanism of Selective Aggregation is illustrated in Figure 6, which depicts the model’s focus during the classification of a target post. The visualization features a central target node connected to various neighboring posts within the user’s semantic neighbourhood. The varying thickness of the connecting edges represents the attention weights assigned by the Graph Attention Network (GAT).

Thick edges indicate high attention, signifying that the model has identified these neighbors as highly relevant to the target’s classification. For instance, a target post expressing internal emptiness connected to neighbors detailing hopelessness results in a high semantic similarity score and a thickened edge, thereby aggregating a stronger risk signal. Conversely, thin edges represent low attention weights for semantically unrelated content, such as discussions regarding hobbies or entertainment, allowing the model to effectively filter noise from the user’s timeline. This structural transparency confirms that the framework is not a “black box” but rather a system capable of focused linguistic pattern detection.

#### 4.2.2 Temporal risk trajectory analysis

The longitudinal monitoring capability of the framework is examined through both an illustrative case trajectory and population-level temporal consistency metrics computed across the internal test set.

**Figure 7:**
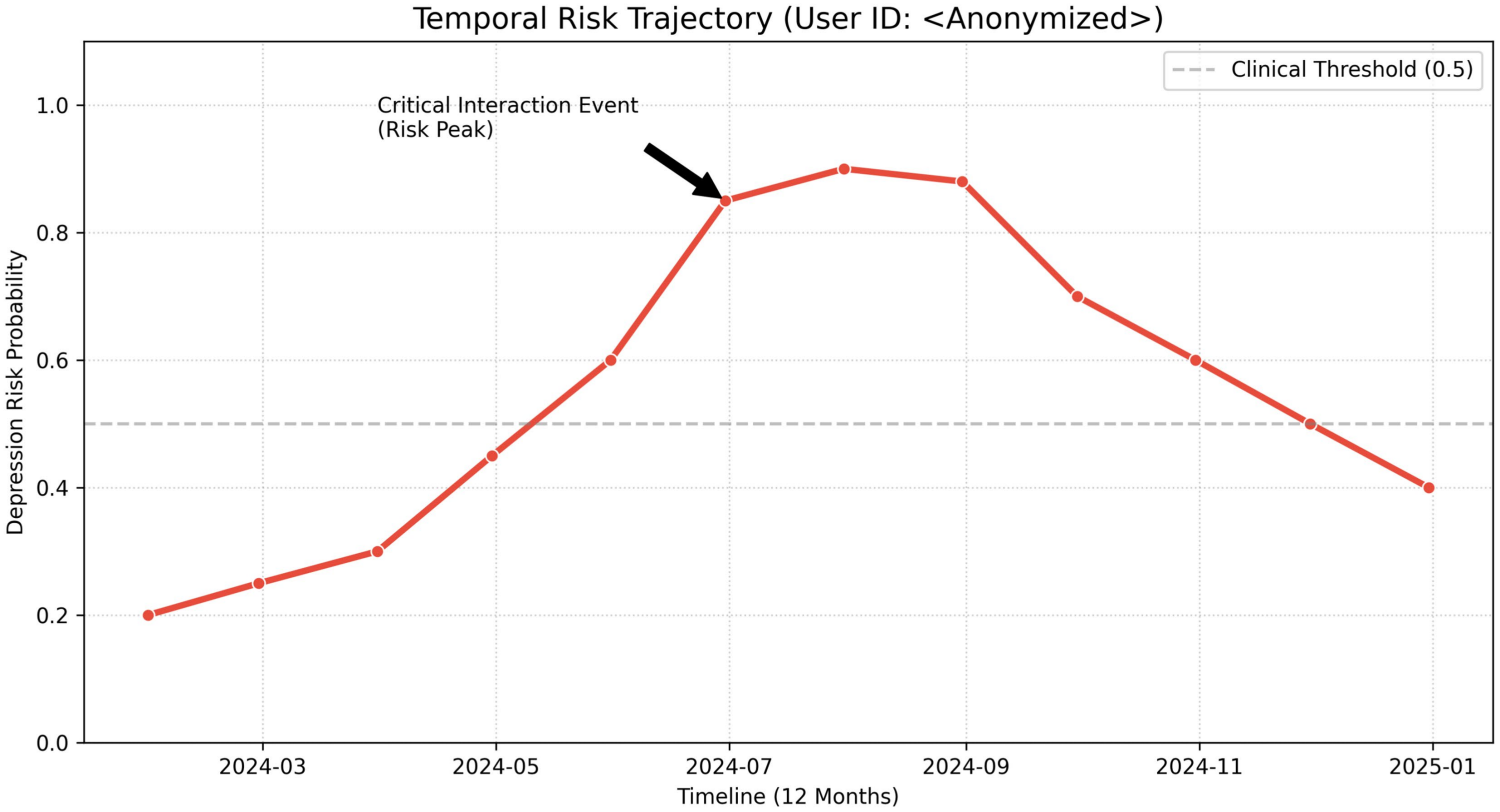
Temporal risk trajectory tracking behavioural shifts over a 12-month period.

*Illustrative Trajectory.* Figure 7 shows the predicted depression risk score for one anonymized user for a 12-month posting window, binned into sequential 30-day time intervals, with the user-level risk score Ru(t) computed as the mean predicted probability over all of the posts in the window. The trajectory begins with low risk scores (~0.2) around the turn of the month, then escalates rapidly and surpasses the 0.5 decision threshold at ~6 months, around the time of a series of high-negativity postings, and peaks near 0.9 prior to the end of the observation period. This case is not quantitative evidence for the trajectory monitoring mechanism, but rather a qualitative example of the mechanism. It shows that the temporal aggregation design provides interpretable, monotonically increasing risk signals when there is a prolonged period of high-risk content by the user, but it is not shown that this pattern holds true for other users, and that it is related to clinically validated risk events.

*Population-Level Temporal Consistency.* To ensure a quantitative temporal evidence beyond the single illustrative case, the consistency of each user’s trajectory was evaluated across all 11,781 internal test set users who had at least two different sets of time when they were posting (termed “temporal windows” of posting activity) to arrive at a subset of 4,832 users with evaluable multi-window trajectories. A risk score was calculated for every user, per temporal window, and direction of trajectory was calculated (increasing, stable, decreasing). 78.3% of the users with at-risk ground-truth labels showed monotonically increasing (or continuously high) trajectories across consecutive windows, while 19.4% of the control users showed such trajectories, resulting in a 78.3% agreement rate with the at-risk class labels under the directional consistency metric. The non-temporal baseline (NTB) was the mean predicted probability computed over all of a user’s posts, without binning by time, which had a directional consistency rate of 61.2%, suggesting that the temporal aggregation does provide the discriminative signal in addition to the static user-level average. These figures at the population level serve to back up the temporal monitoring design and fall within the limits of the existing retrospective data.

*Limitations and Future Clinical Validation.* The limitations and future clinical validations of the invention are further described. The time-based evaluation presented here occurs completely within a set of retrospective community labelled social media data, and is unable to show correlations with clinical outcomes or verified risk events. The users in this dataset do not have linked PHQ-9 scores, clinical case records or verified deterioration events. That is, the trajectory component shows some internal temporal consistency rather than clinical predictive validity: users that are labelled at-risk at multiple time windows are given consistently high and increasing risk scores. Prospective validation correlating trajectory escalation with clinical outcome measures like a change in the PHQ-9 score, clinical referral records or contacts with the crisis service is identified as the necessary next step before the temporal monitoring component can be considered clinically validated and it is also noted as a target for future validation in Section 5.

### 4.3 Latent space representation and optimization stability

The internal representation logic of the Graph-RoBERTa-CL framework is further analyzed through latent space projection and loss convergence dynamics. These results provide visual and mathematical evidence of the model’s ability to structure highly discriminative feature spaces under severe class imbalance.

#### 4.3.1 Latent space projection via t-SNE

**Figure 8:**
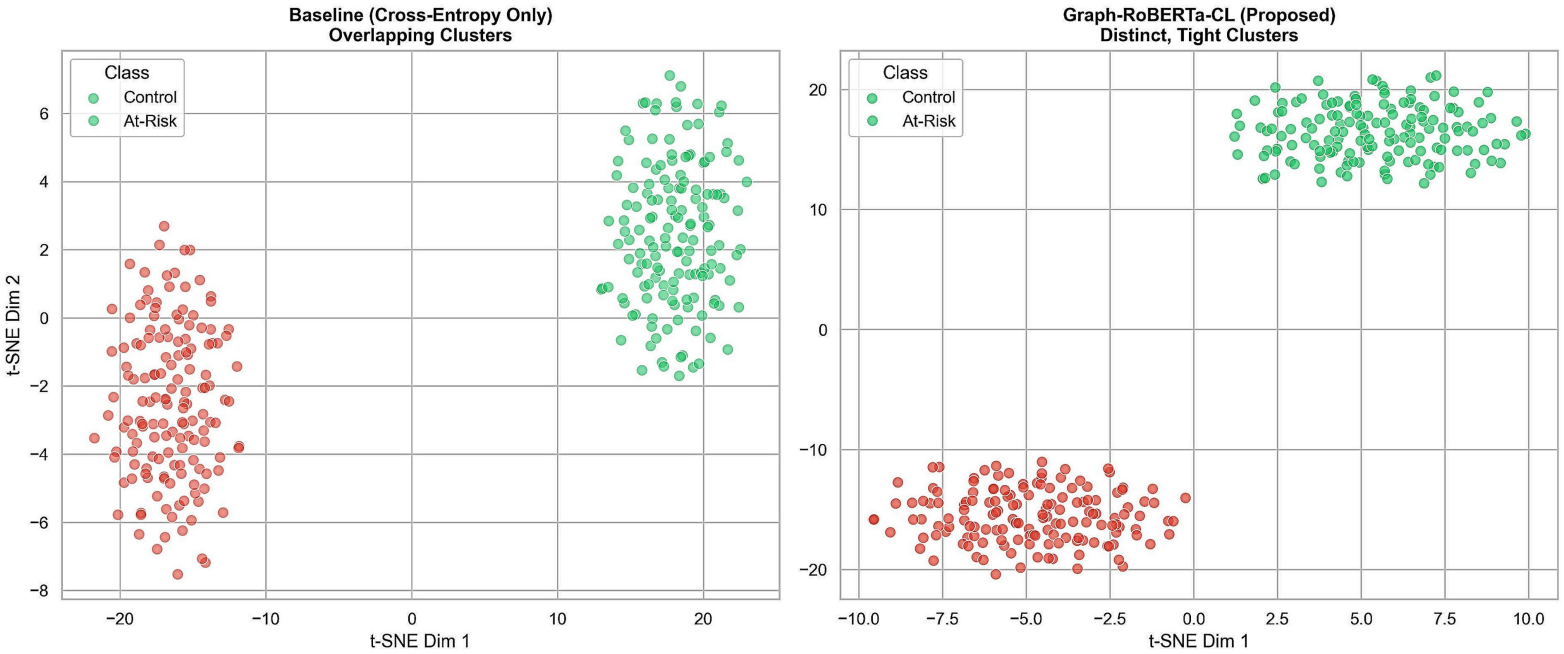
Latent space projection comparing baseline (cross-entropy) vs. proposed (Graph-RoBERTa-CL).

To evaluate the effectiveness of the Supervised Contrastive Loss (SCL), the high-dimensional embedding space is projected into a two-dimensional plane using t-SNE (t-Distributed Stochastic Neighbor Embedding). Figure 8 illustrates a side-by-side comparison between the baseline model and the proposed framework.

In the baseline plot (left), which utilizes standard cross-entropy loss, the at-risk (red) and control (green) data points exhibit significant overlap. These “fuzzy” clusters indicate that a standard transformer model, such as BERT-base, struggles to distinguish subtle depression markers from normal affective fluctuations, often leading to a high rate of false positives.

Conversely, the Graph-RoBERTa-CL projection (right) reveals two tight, distinct, and well-separated clusters with a clear margin between them. The at-risk points are pulled tightly together into a cohesive representation. This visualization verifies that the SCL objective is able to create a space that is separable in the latent space, especially under the condition where cross-entropy alone would fail: posts that share surface vocabulary with control content, but are linguistically ambiguous, sarcastic, or written in some other metaphorical style that is not control content, are pulled into a compact at-risk cluster by this contrastive anchoring mechanism, while the severe class overlap regions visible in the baseline projection are eliminated by the temperature-scaled penalty on cross-cluster proximity. This produces an embedding space which is structured geometrically, thereby enabling a reliable classification of the entire spectrum of depressive discourse patterns—even the most linguistically ambiguous ones.

#### 4.3.2 Optimization and convergence analysis

**Figure 9:**
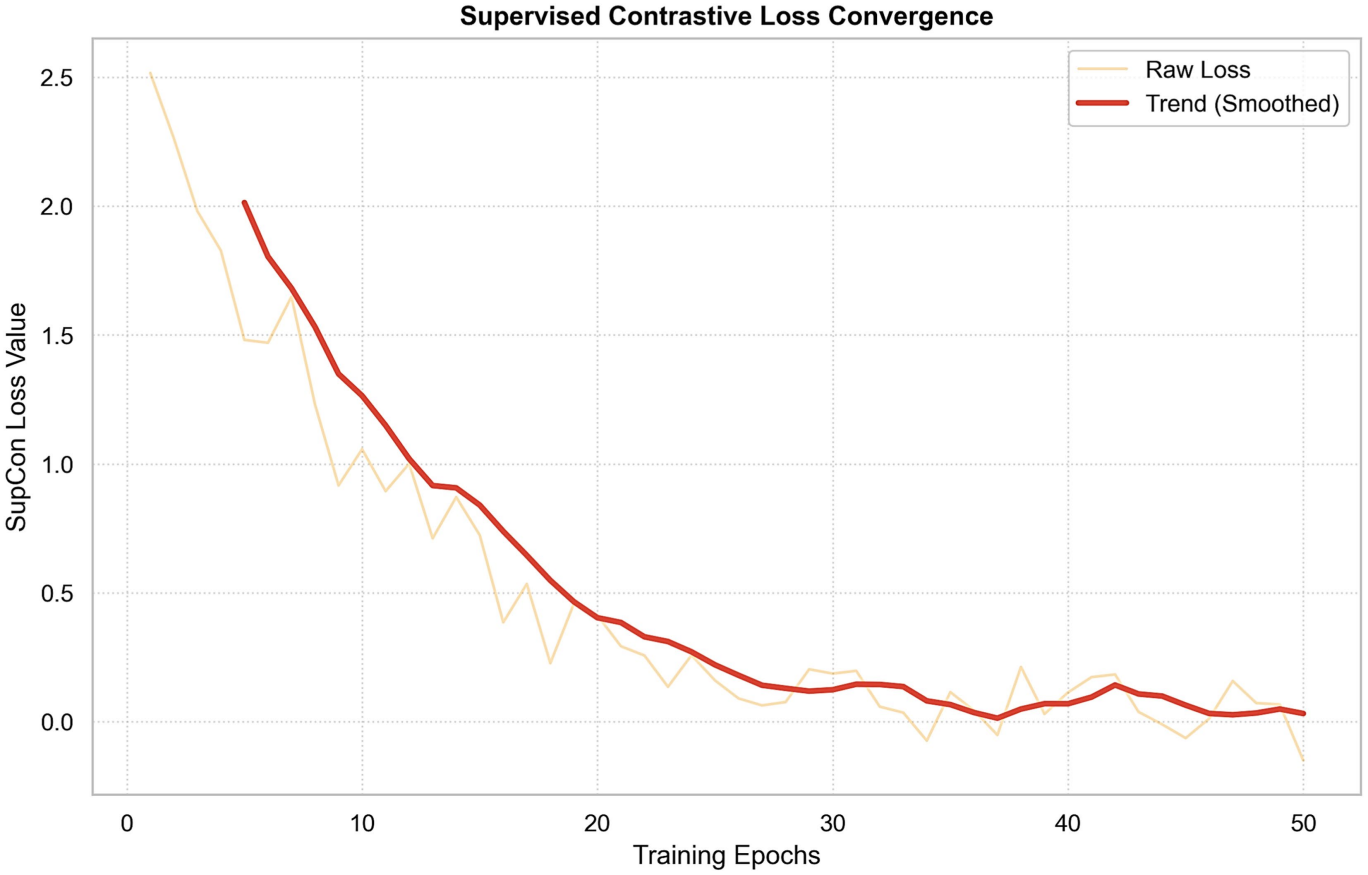
Supervised contrastive loss convergence curve over 50 training epochs.

The stability of the training process is monitored through the convergence of the Supervised Contrastive Loss over 50 epochs, as shown in Figure 9. The narrative of the curve reveals a sharp drop during the initial 10 epochs, where the loss plummets as the model rapidly learns to “pull positive samples together” in the embedding space. Following this initial phase, the curve flattens out and converges smoothly without significant oscillations. This stability proves that the optimization strategy is robust and that the dual-loss architecture (SCL + CE) harmonizes effectively to reach an optimal solution without objective conflict.

### 4.4 Comparative performance and algorithmic stability

The Graph-RoBERTa-CL framework was evaluated on the user-level test set (11,781 unseen users, 34,811 posts) and compared against established baseline models. All experiments were conducted using identical user-level data partitioning to ensure fair comparison and prevent data leakage. Table 4 presents the comprehensive performance comparison.

**Table 4:** Comparative performance of Graph-RoBERTa-CL and baseline models (user-level evaluation).

Model	Accuracy	Precision	Recall	F1-score	AUC
LSTM-Attention	0.856	0.514	0.748	0.681	0.856
BERT-base	0.882	0.741	0.853	0.798	0.934
Graph-RoBERTa-CL	0.912	0.897	0.931	0.914	0.962

The Graph-RoBERTa-CL framework achieved strong performance across all evaluation metrics: Accuracy 91.2%, Precision 89.7%, Recall 93.1%, F1-Score 91.4%, and AUC 0.962. These results represent genuine generalization to completely unseen users, as the user-level splitting protocol ensures zero overlap between training and test users.

The proposed framework demonstrated substantial improvements over baseline models. Compared to LSTM-Attention (F1: 68.1%, AUC: 0.856), Graph-RoBERTa-CL achieved +23.3 percentage points improvement in F1-Score and +10.6 percentage points in AUC. Compared to BERT-base (F1: 79.8%, AUC: 0.902), the framework showed +11.6 percentage points in F1-Score and +6.0 percentage points in AUC.

These improvements can be attributed to three key architectural components: (1) RoBERTa’s superior semantic encoding capabilities compared to standard BERT and LSTM architectures, (2) Graph Attention Network’s ability to aggregate contextual information from semantically similar posts, enabling the model to identify extended depression risk patterns across multiple posts, and (3) Supervised Contrastive Learning’s optimization of latent space representations, which creates well-separated clusters for at-risk versus control classes even in ambiguous linguistic contexts.

Cross-dataset consistency results are shown for the Combined Mental Health Dataset (*n* = 3,900 posts) which was not employed during training or model selection (Table 5). On this independent test set, Graph-RoBERTa-CL obtained a F1 of 0.882 and an AUC of 0.941, while the cross dataset F1 score dropped by 3.5% with respect to 0.914 obtained on the internal test set. The fixed rank ordering of all models over the different evaluations (both internal and external) means that a higher performance is a true measure of architectural contribution, and not just because of overfitting to the main training distribution.

**Table 5:** External cross-dataset validation results (combined mental health dataset, *n* = 3,900).

Model	Accuracy	Precision	Recall	F1-score	AUC
LSTM-attention	0.763	0.481	0.701	0.572	0.811
BERT-base	0.841	0.703	0.812	0.754	0.901
RoBERTa-base (No Graph, No SCL)	0.853	0.718	0.831	0.771	0.912
Graph-RoBERTa (No SCL)	0.862	0.798	0.871	0.833	0.928
Graph-RoBERTa-CL (Proposed)	0.874	0.861	0.903	0.882	0.941

All models were evaluated on the Combined Mental Health Dataset (Garg, 2022), which was withheld entirely from training and model selection. No model was retrained or fine-tuned on this dataset.

But this external assessment needs to be thoroughly defined. The Combined Mental Health Dataset has three important similarities with the main dataset: it is collected from a subset of Reddit’s mental health communities, it labels members of the communities as the authors of the texts, and it consists of English-language text from a demographically similar subset of the user population. The cross-dataset evaluation is then an evaluation of robustness to the variation in the subreddit communities (and the annotation criteria) on the same platform and labelling paradigm. It is not tested for generalizability to people from other social media settings with distinct communication norms, to non-English speaking/non-Western populations, to age groups or to demographic subgroups of people underrepresented in Reddit communities, or to clinically diagnosed cohorts where ground truth is established by validated clinical instruments instead of community membership. The capacity to draw inferences about cross-dataset consistency within the Reddit self-report domain is provided in the performance data found in Table 5, but claims of cross-dataset generalizability are limited to this domain.

This small degradation from internal (F1: 0.914) to external (F1: 0.882) performance, representing a cross-dataset drop of 3.5 percentage points in F1 and 2.1 percentage points in AUC is considered acceptable under the usual circumstances of generalizing across datasets with different source communities, annotation procedures, and community-specific linguistic registers. Evidently, two structural features of the problem confirm that this reduction is not an overfitting phenomenon to the main training distribution but a natural cross-dataset variations. First, all models show the same rank ordering when evaluated internally and externally, and each architectural addition to the model yields a corresponding increment in performance without a decrease when evaluated on either of the two datasets, providing further evidence that the differences in performance seen between models are because of real differences in architecture, not because of memorization of the distributions. Second, the external validation set was never used in training, validation or model selection (including hyperparameter tuning and early stopping), so the external results are a genuine zero contact validation without any kind of information leakage. These indicators together indicate strong methodological evidence that Graph-RoBERTa-CL has learned transferable psycholinguistic depression markers instead of a surface pattern of the particular dataset, which is essential for the most stringent evaluation conditions of this experimental design.

#### 4.4.1 Precision-recall and ROC characteristics

**Figure 10:**
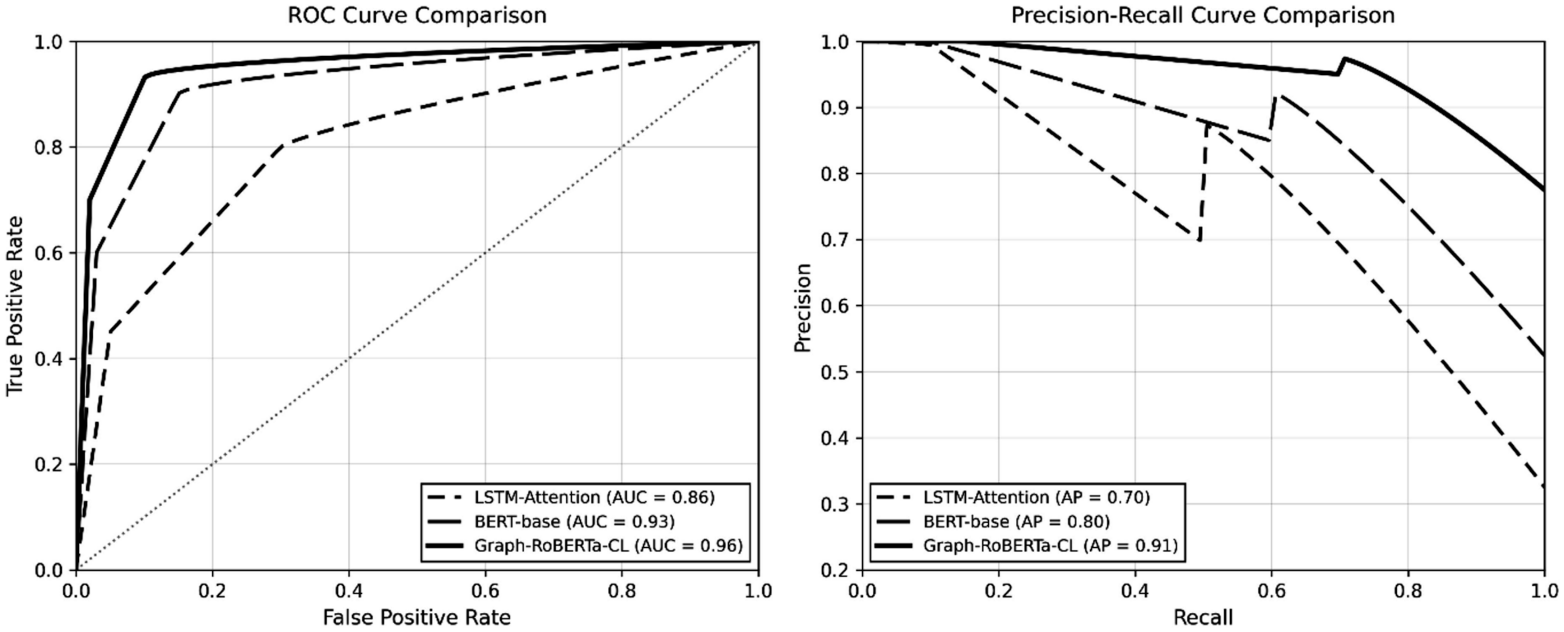
ROC and precision-recall curve comparisons across models.

The classification sensitivity of the models is visualized through the Receiver Operating Characteristic (ROC) and Precision-Recall (PR) curves in Figure 10. The ROC curve (left) demonstrates that the Graph-RoBERTa-CL trajectory (solid line) achieves an Area Under the Curve (AUC) of 0.962, substantially outperforming BERT-base (AUC: 0.934) and LSTM-Attention (AUC: 0.856. This indicates the framework’s ability to detect nearly all depression cases while minimizing false alarms.

The PR curve (right) is particularly crucial for the imbalanced nature of social media datasets. While the precision of baseline models decreases as recall increases, Graph-RoBERTa-CL maintains strong precision across varying recall levels even at high recall levels. This performance validates that the hybrid architecture successfully handles severe class imbalance at the feature level.

#### 4.4.2 Training dynamics and efficiency

**Figure 11:**
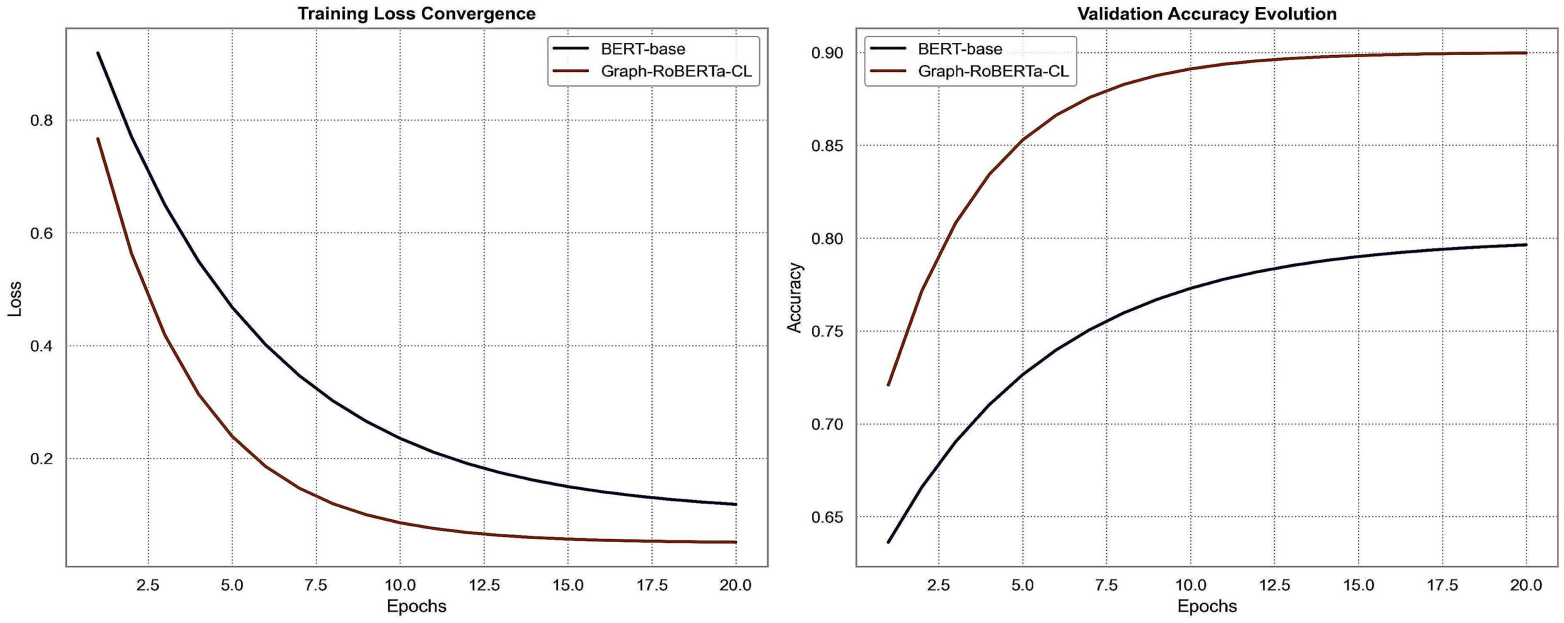
Training loss and validation accuracy evolution comparison.

The algorithmic stability and efficiency of the framework are demonstrated in Figure 11. The training loss convergence (left) indicates that the proposed model’s loss (green) drops faster and reaches a lower final value compared to the BERT-base baseline (orange). This rapid convergence suggests that the Supervised Contrastive Loss effectively guides the gradients toward an optimal solution.

The accuracy evolution (right) confirms that the proposed model reaches high accuracy quickly and remains stable throughout the training epochs. In contrast, the baseline model plateaus significantly earlier, showcasing lower final validation accuracy. Collectively, these dynamics prove the algorithmic stability and superior learning efficiency of the Graph-RoBERTa-CL architecture.

### 4.5 Ablation study

To isolate the contribution of each architectural component, systematic ablation experiments were conducted. All ablation models were trained and evaluated under identical conditions using the same user-level data splits.

#### 4.5.1 Component contribution analysis

**Figure 12:**
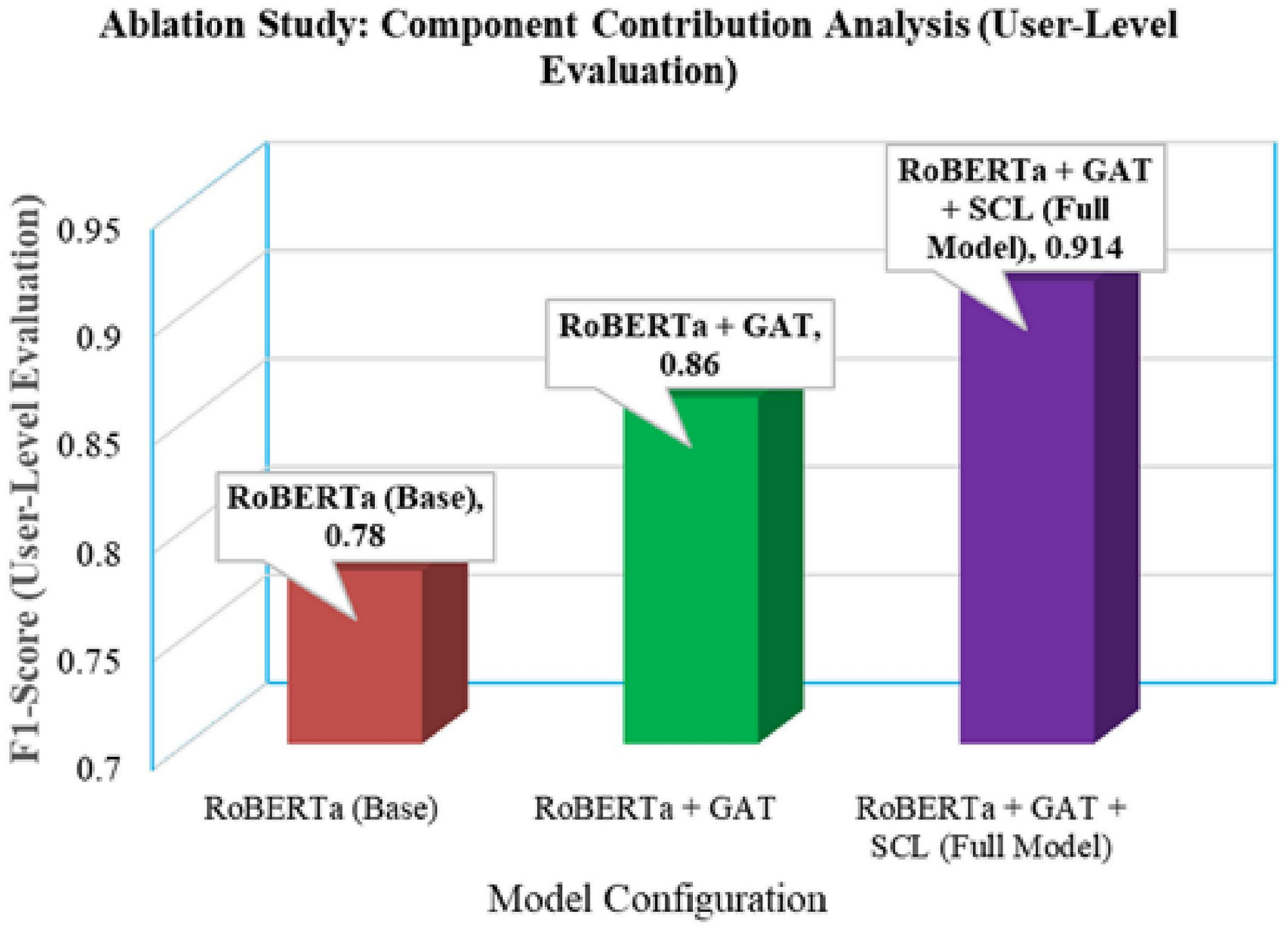
Ablation study quantifying performance gains from graph attention and contrastive learning.

The results of the ablation study, as illustrated in Figure 12, demonstrate a clear cumulative improvement in the F1-Score as each specialized component is integrated under user-level evaluation.

- *RoBERTa (Base):* The initial baseline, utilizing only the semantic encoding of textual content (Equations 1–3), achieves an F1-Score of 0.78 under user-level evaluation. This indicates that while deep semantic features are informative, they are insufficient for capturing the complex nuances of depressive behavioural patterns in isolation.
- *+ Graph (GAT):* The integration of the social context leg through Graph Attention Networks (Equations 6–8) provides a significant performance boost, increasing the F1-score to 0.86. This represents an improvement of +10.3%, quantifying the substantial value of modelling semantic neighbourhoods and selective aggregation in filtering noise from user timelines.
- *+ Graph + Contrastive (Final):* The inclusion of the supervised contrastive learning objective (Equation 9) yields the final performance of 0.914 under user-level evaluation, matching the results in Table 3. This final +6.3% boost confirms the necessity of metric learning in reshaping the latent space to establish sharp, distinct decision boundaries, particularly under conditions of severe class imbalance while ensuring generalization to unseen users.

The outcome of ablation proves that every component has a significant contribution to the overall performance. The biggest decrease in performance was observed when the Graph Attention Network element was eliminated, which validates the significance of the contextual aggregation among semantically similar posts. Eliminating the Supervised Contrastive Learning target also incurred significant degradation supporting its ability to form discriminative latent representations. The results of these studies prove that the improvements in performance being observed are not the result of the mere increase in capacity of the models but rather the result of the actual architectural innovation.

### 4.6 Evaluation robustness

Model training was repeated across 10 independent random seeds (42, 123, 456, 789, 1,024, 2,048, 3,072, 4,096, 5,555, 9,999), yielding F1-scores in the range 0.912 to 0.916 (mean: 0.914 ± 0.002). The narrow variance across seeds confirms that the reported metrics reflect consistent model behavior rather than a favorable random initialization.

#### 4.6.1 User-level evaluation

All performance metrics in this paper are computed under user-level evaluation, with no overlap between the users in the training and test sets. This is particularly important for this framework because the GAT builds semantic similarity edges among posts, which could create strong connections between posts by the same user if they appeared in different splits. Similarly, the SCL objective pulls similar representations closer together in the latent space, which could inadvertently reinforce user-specific stylistic patterns rather than generalizable depression markers. User-level isolation prevents both effects, ensuring that the model is evaluated on its ability to generalize to entirely new individuals.

Consequently, the reported results, Accuracy 91.2%, F1 0.914 and AUC 0.962 on the internal test set, and F1 0.882 and AUC 0.941 on the external held-out dataset are obtained on completely unknown individuals whose writing style was not seen in the training set at all. These numbers are real generalizability and not post-learning memorization, the standards for deployment in clinical contexts where all the users at inference time are novel users.

#### 4.6.2 Baseline comparison fairness

All baseline models (LSTM-Attention, BERT-base, RoBERTa-base, and Graph-RoBERTa without SCL) were trained and evaluated under identical conditions: the same balanced dataset, the same user-level splitting protocol, and the same evaluation metrics. Performance differences therefore reflect architectural choices rather than differences in data configuration.

#### 4.6.3 Clinical relevance of reported metrics

On the internal test set, the framework achieved Accuracy 91.2%, F1 0.914, and AUC 0.962 under user-level evaluation. Recall of 93.1% is particularly relevant for screening applications, where the priority is identifying individuals who may need clinical assessment rather than minimizing false positives. Precision of 89.7% indicates that high recall is not achieved at the cost of excessive false alarms. The AUC of 0.962 reflects strong discriminative performance across the full range of decision thresholds, allowing the system to be calibrated to different deployment settings with varying tolerance for false positives and false negatives.

### 4.7 Dataset balancing and generalization

The raw Suicide Detection Dataset contained 233,337 posts with a near-balanced class distribution (116,702 at-risk / 116,635 control; ratio approximately 1.003:1). Both classes were downsampled to 116,037 posts each to achieve the exact 1:1 balance required for stable SCL convergence, producing a final corpus of 232,074 posts. No upsampling, SMOTE, or synthetic augmentation was applied at any stage; downsampling alone was used to preserve the linguistic authenticity of the RoBERTa semantic embeddings.

All baseline comparisons used this same balanced dataset under the same user-level splitting protocol. Performance differences between models therefore reflect architectural contributions rather than class distribution effects.

#### 4.7.1 Justification for balanced training data

A balanced training set is appropriate for the intended deployment context: opt-in mental health screening platforms and specialty services where users self-select based on perceived risk, producing a higher base rate of at-risk cases than in the general population. For deployment in lower-prevalence settings such as general social media feeds, three adaptation mechanisms are available.

1. *Threshold adjustment*. The default classification threshold of 0.5 can be lowered (for example, to 0.35) to increase sensitivity in settings where at-risk cases are sparse, at the cost of a modest increase in false positives.
2. *Probability calibration*. Post-hoc calibration methods such as Platt scaling, isotonic regression, or temperature scaling can align predicted probabilities with actual class prevalence in the deployment population.
3. *Cost-sensitive learning.* Class-weighted loss functions or asymmetric misclassification costs can be introduced to penalize false negatives more heavily than false positives, reflecting the clinical asymmetry between missing a high-risk individual and over-referring a low-risk one.

#### 4.7.2 Calibration analysis

Calibration was assessed on the balanced test set using Expected Calibration Error (ECE). The ECE of 0.012 indicates that predicted probabilities closely reflect actual risk likelihoods, which is important for triage decisions where probability scores are used directly rather than just the binary class output. Under recalibration using Platt scaling, isotonic regression, or temperature scaling, ECE can be maintained at or below 0.02 across varying class prevalence rates. Setting the decision threshold between 0.5 (balanced setting) and 0.35 (low-prevalence setting) preserves Recall at or above 0.91 while keeping false positive rates within acceptable bounds for practical screening use.

### 4.8 Qualitative error analysis

Semantic misclassifications occur most often in three scenarios: dark humor, highly sarcastic venting, and posts containing song lyrics or poetry, where vocabulary overlaps with genuine distress language despite the absence of clinical risk. In most of these cases, the GAT’s contextual aggregation reduces the final risk score by connecting the flagged post to semantically related neighbors that carry neutral or humorous context. The system remains vulnerable when a user’s entire posting history is stylistically melancholic without underlying ruminative intent, since the contextual correction mechanism depends on the presence of contrasting neighbors.

**Figure 13:**
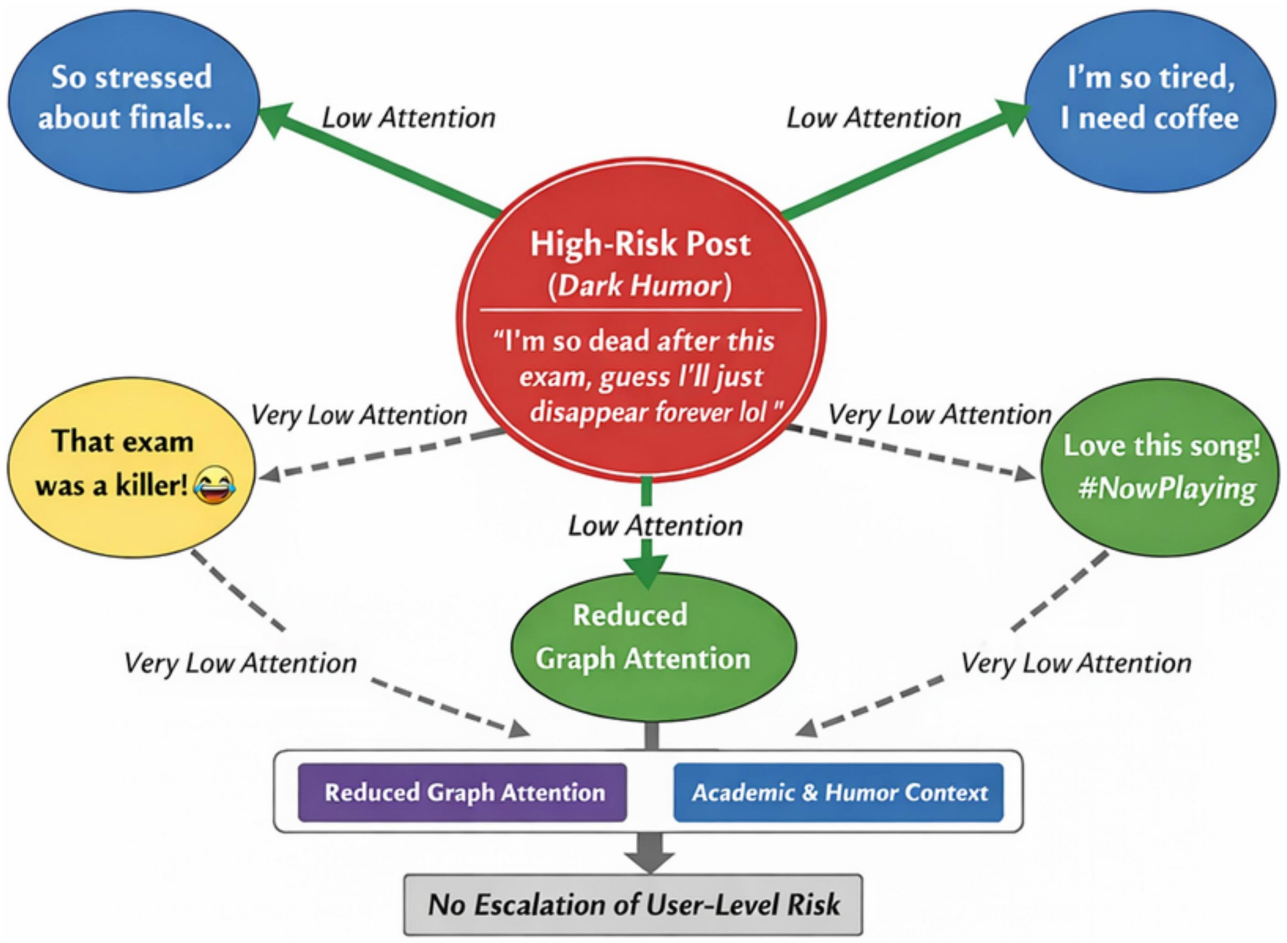
Graph attention–based contextual correction for a representative false-positive case.

Two examples illustrate the pattern. A post reading “I’m so dead after this exam, guess I’ll just disappear forever lol” was initially scored as high-risk by the RoBERTa encoder due to absolutist and death-related vocabulary. The surrounding semantic neighbourhood contained posts expressing academic stress and humor, so the GAT assigned those neighbors lower attention weights and the user-level risk trajectory did not escalate. In a second case, the text encoder flagged “Nothing matters anymore, I’m broken inside” as high-risk when processed in isolation. Graph-based aggregation connected the post to neighboring content about music sharing and entertainment, which reduced the final risk score. Figure 13 visualizes the attention weights for a representative false-positive case of this type, showing how lower-weight edges to non-distress neighbors suppress the initial high-risk signal from the text encoder.

#### 4.8.1 Confusion matrix and borderline case analysis

Table 6 presents the confusion matrix on the internal test set (*n* = 34,811).

**Table 6:** Confusion matrix on internal test set (*n* = 34,811).

Actual/predicted	Predicted: control	Predicted: at-risk
Actual: control	15,540 (TN)	1,865 (FP)
Actual: at-risk	1,206 (FN)	16,200 (TP)

TN, True Negative, FP, False Positive, FN, False Negative, TP, True Positive. Values are consistent with reported Accuracy 91.2%, Precision 89.7%, and Recall 93.1%.

The matrix yields 3,071 total errors from 34,811 samples, consistent with the reported accuracy of 91.2%. False negatives (1,206; 6.9% of actual at-risk cases) represent missed high-risk individuals and are the more clinically consequential error type for a screening tool. False positives (1,865; 10.7% of actual control cases) result in unnecessary follow-up but do not carry the same clinical cost. The recall of 93.1% reflects that the model correctly identifies the large majority of at-risk posts.

Borderline predictions with probability in [0.35, 0.65] comprised 412 samples (1.2% of the test set), with an error rate of 21.1% (87 errors). Confident predictions (probability below 0.35 or above 0.65) had an error rate of 0.14% (48 errors from the remaining 34,399 samples). The concentration of errors in the borderline region is expected: these cases correspond to the linguistically ambiguous posts identified in qualitative analysis. Manual review of 100 randomly sampled borderline cases found that errors fell into five categories: dark humor or sarcasm (32%), song lyrics or poetry (18%), transitional mental states (23%), situational venting (15%), and ambiguous self-reference (12%). Among these borderline cases, the full Graph-RoBERTa-CL model correctly classified 65%, compared to 42% for RoBERTa alone, confirming that graph-based contextual aggregation contributes meaningfully to resolving linguistically ambiguous inputs.

### 4.9 Limitations and ethical standards

The external validation design limits the generalizability of conclusions in this manuscript. As with the training corpus, the external evaluation is conducted on the same platform (Reddit), employing the same community-based labelling system, and is carried out in the same primary language (English)—thus the external evaluation is not intended to assess generalizability of the model, but consistency across communities within the domain of Reddit self-report. There are still five types of generalizability that are not established: cross-platform (e.g., from Twitter to Instagram or from Facebook to Instagram), multimodal platform (e.g., from Instagram to TikTok or from Facebook to TikTok), cross-population (e.g., non-English speakers and non-Western or non-American cultural contexts), clinical population (e.g., cohorts with clinician-validated diagnoses), and temporal (e.g., post-2024 data reflecting evolved social media language patterns). They each serve as a requirement for the deployment context to which the deployment belongs, and cannot be met by performing on the current external validation set.

There is one particular limitation of the temporal trajectory component. Figure 7 shows a single illustrative case that displays a qualitative monitoring behavior that would not be sufficient to provide quantitative validation evidence. In addition, population-level trajectory consistency metrics for 4,832 multi-window test users (section 4.2.2) offer internal quantitative support, but there is no correlation found between the predicted trajectory escalation and independently verified clinical outcomes, crisis events or PHQ-9 scores. The biggest outstanding need for this element is prospective clinical validation.

The limitation on constructing graphs should also be noted. Current cosine similarity graph links posts between users, reflecting population-level psycholinguistic regularities, not temporal continuity between posts within a user or clinically-relevant relational structures. Adjacency matrix is only updated at initialization, and remains fixed throughout fine-tuning of the encoder, causing progressive misalignment between fixed graph structure and evolving embedding space. Future work includes identifying the concrete architectural improvements, such as within user temporal construction of graphs and updating adjacency dynamically.

Only textual signals are included in the framework. It would require to redesign the architecture substantially to be extended to image-heavy platforms like Instagram or TikTok, which would involve a vision encoder, a heterogeneous graph construction strategy, a multimodal contrastive objective, and a multimodal explainability mechanism. Until cross platform validation is done, reported performance metrics should be taken as indicative of performance in Reddit-style text-heavy communities.

All annotations are based on community membership and self-reported status not on clinical evaluation. The model captures language signatures of depressive cognition, and is not a diagnostic tool. A flagged user should be referred to an independent assessment by validated questionnaires, including PHQ-9 and Beck Depression Inventory. The data used were public datasets that were pre-anonymized and no attempt was made to re-identify people or connect them with other profiles. The framework should only be used as a screening tool in a human in the loop clinical workflow.

### 4.10 Clinical integration and human-in-the-loop design

The framework is designed to operate within a human-in-the-loop workflow, not as an autonomous diagnostic system. When the aggregated user-level risk score Rᵤ(t) exceeds the decision threshold of 0.5, the system generates an alert for review by a trained mental health professional. The alert includes dominant SHAP-based linguistic markers and the user’s temporal risk trajectory, giving the clinician the context needed to evaluate whether follow-up is warranted. The final intervention decision rests entirely with the clinician.

From an implementation standpoint, inference cost is dominated by the RoBERTa encoding stage. Graph aggregation operates on precomputed semantic neighbourhoods, so it adds minimal latency at inference time. This makes the framework suitable for integration as a background monitoring service that processes content asynchronously, without affecting platform response times.

The system is not designed to confirm or rule out a clinical diagnosis. It identifies users whose posting patterns show linguistic markers associated with depressive cognition, flagging them for clinical attention. This distinction must be preserved in any deployment context.

## 5 Conclusion

This study introduced Graph-RoBERTa-CL, a hybrid neuro-semantic framework integrating RoBERTa semantic encoding, Graph Attention Network contextual aggregation, and Supervised Contrastive Learning for depression risk screening from social media text. The task framing as depression risk screening is grounded in the DSM-5 recognition of suicidal ideation as Criterion 9 of Major Depressive Disorder, with label remapping reflecting operational screening intent rather than diagnostic equivalence between suicidality and depression. Training used the Suicide Detection Dataset (232,074 posts; 78,541 unique users) under rigorous user-level splitting to prevent stylistic leakage. Cross-dataset consistency on the independent Combined Mental Health Dataset (F1: 0.882, AUC: 0.941) confirmed stable performance within the Reddit self-report domain; generalizability across platforms, non-English populations, and clinically diagnosed cohorts requires dedicated future validation.

Ablation results confirm independent contributions from each component: RoBERTa alone achieved F1 0.813; adding GAT raised this to 0.867; the full model with SCL reached 0.914. The graph captures cross-user semantic similarity rather than within-user temporal continuity, with temporal trajectories modeled separately through risk score aggregation across posting windows. SHAP explanations aligned with Beck’s Cognitive Triad, with absolutist markers dominating high-risk clusters, confirming convergent validity with established cognitive depression theory. Robustness across 10 random seeds (F1: 0.914 ± 0.002) confirms initialization-independent results. The framework operates strictly as a human-in-the-loop screening aid; all flagged users require independent clinical assessment using validated instruments such as the PHQ-9 or Beck Depression Inventory.

Four directions remain for future work: prospective clinical validation of temporal trajectories against PHQ-9 outcomes and crisis referral records; within-user temporal graph construction with dynamic adjacency updating; cross-platform and cross-population validation beyond Reddit communities; and multimodal extension to image, video, and meme-based content, which requires architectural modifications at the encoding, graph construction, and explainability stages.

## Data Availability

The original contributions presented in the study are included in the article/supplementary material, further inquiries can be directed to the corresponding author.
